# External validation of established clinical risk scores for cancer-associated venous thromboembolism in a Brazilian registry

**DOI:** 10.1007/s11239-026-03303-6

**Published:** 2026-05-18

**Authors:** Márcia Fayad Marcondes de Abreu, Mohamad Al Bannoud, Sandra Martins, Stephany C. Huber, Beatriz de Moraes Martinelli, Maria Carmen GL Fernandes, Júlio C Teixeira, José BC Carvalheira, Carmen SP Lima, Nelson A Andreollo, Maurício Etchebehere, Lair Zambon, Ubirajara Ferreira, Alfio José Tincani, Antônio Santos Martins, Cláudio SR Coy, José CT Seabra, Ricardo K Mussi, Helder Tedeschi, Daniel Dias Ribeiro, Cyrillo Cavalheiro Filho, Tiago Dias Martins, Gabriel Yoshiaki Ottaiano, Rubens Maciel Filho, Silmara Aparecida de Lima Montalvão, Joyce Maria Annichino-Bizzacchi

**Affiliations:** 1 Celso Pierro Hospital and Maternity, Angiology and Vascular Surgery Service, Campinas, São Paulo, Brazil; 2 Center for Thromboembolic Diseases (CDT), Hemocentro Unicamp, State University of Campinas (UNICAMP), Campinas, São Paulo, Brazil; 3 School of Chemical Engineering, Laboratory of Optimization, Design, and Advanced Control, State University of Campinas (UNICAMP), Campinas, São Paulo, Brazil; 4 Department of Chemical Engineering, Institute of Environmental, Chemical and Pharmaceutical Sciences, Federal University of São Paulo (UNIFESP), Diadema, São Paulo, Brazil; 5 Women’s Comprehensive Health Care Center (CAISM), State University of Campinas (UNICAMP), Campinas, São Paulo, Brazil; 6 Hospital das Clinicas, Faculty of Medical Sciences, State University of Campinas (UNICAMP), Campinas, São Paulo, Brazil; 7 Hospital das Clínicas, Federal University of Minas Gerais (UFMG), Belo Horizonte, Minas Gerais Brazil; 8 Instituto do Coração do Hospital das Clínicas da FMUSP, University of São Paulo (USP), São Paulo, São Paulo, Brazil

**Keywords:** Cancer-associated thrombosis, Venous thromboembolic risk assessment, Khorana score validation, Biomarker-based risk stratification, Brazilian cancer cohort

## Abstract

Cancer-associated thrombosis (CAT) is a relevant cause of morbidity and mortality in oncology patients. Although several venous thromboembolism (VTE) risk assessment models (RAMs) are recommended to guide thromboprophylaxis in ambulatory cancer patients, their performance varies across populations. Data from Brazilian cohorts are limited, and the real-world performance of these models in this setting remains unclear. We conducted a prospective external validation study of four established VTE RAMs—Khorana, PROTECHT, CONKO, and Vienna CATS—in ambulatory cancer patients from a Brazilian tertiary-care registry. A total of 803 adult patients with complete data were followed for 12 months for objectively confirmed symptomatic VTE. Predictors included baseline variables from each RAM, applied according to their original definitions. Discrimination was assessed using the area under the receiver operating characteristic curve (AUC), with 95% confidence intervals (CI) obtained by bootstrap resampling. Vienna CATS was evaluated in a biomarker-defined subcohort of 470 patients. During follow-up, 36 of 803 patients (4.5%) developed VTE. The Khorana (AUC 0.567; 95% CI 0.462–0.672), PROTECHT (AUC 0.575; 95% CI 0.470–0.680), and CONKO (AUC 0.567; 95% CI 0.464–0.671) scores showed limited and comparable discrimination, with sensitivities of approximately 30% at the conventional high-risk threshold (≥3 points). In the Vienna CATS subcohort, 24 of 470 patients (5.1%) developed VTE. Vienna CATS demonstrated modest but statistically significant discrimination (AUC 0.672; 95% CI 0.559–0.779) over chance. However, pairwise comparisons using DeLong’s test showed no significant differences in AUC between the clinical-only scores and the same subcohort. Additional analyses suggested heterogeneity in risk across component combinations despite equal point weighting. Among patients with very-high-risk tumors plus one additional component, VTE incidence varied substantially depending on the specific factor present, ranging from 0% for BMI to 38.5% for low hemoglobin. Overall, current RAMs demonstrated limited sensitivity for identifying patients who developed VTE in this Brazilian cohort. These findings indicate that RAMs alone may be insufficient to guide thromboprophylaxis decisions in this setting, particularly given their limited sensitivity and the occurrence of VTE events among patients classified as low risk. Furthermore, the results support the need to develop locally validated models for Brazilian patients, as well as to explore novel biomarkers, alternative predictor thresholds, and potentially nonlinear approaches to variable weighting.

**Graphical abstract:**

**Figure:**
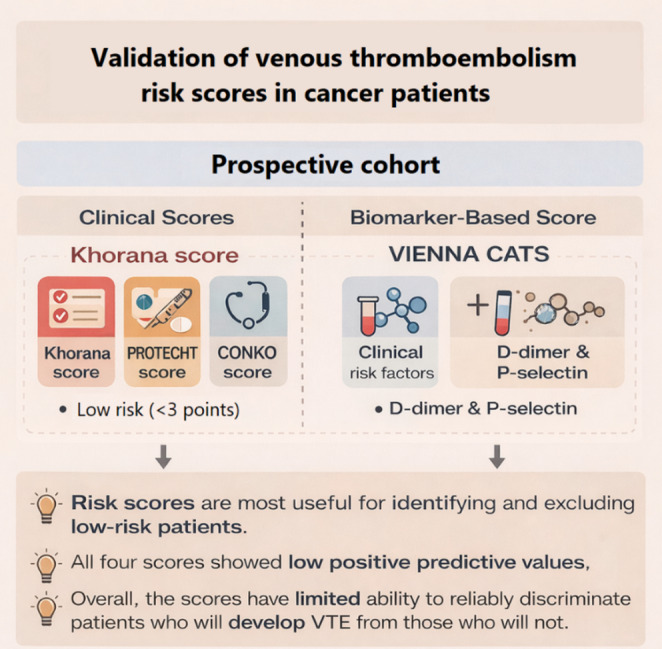

**Supplementary Information:**

The online version contains supplementary material available at 10.1007/s11239-026-03303-6.

## Key points

• This prospective cohort enabled external validation of clinical-only (Khorana, PROTECHT, CONKO) and biomarker-based (Vienna CATS) VTE risk scores in Brazilian cancer outpatients.

• Clinical-only scores demonstrated limited discrimination (AUC ≈ 0.57) and low sensitivity (~ 30%) at conventional high-risk thresholds.

• Vienna CATS showed numerically higher discrimination (AUC 0.672) but was not statistically superior in direct comparisons.

• Substantial heterogeneity in VTE incidence was observed across combinations of equally weighted risk components, suggesting potential non-linearity in thrombotic risk relationships not captured by current scores.

• Future research should integrate biomarkers and nonlinear modeling to develop more accurate, locally applicable VTE prediction tools.

**Supplementary Information:**

The online version contains supplementary material available at 10.1007/s11239-026-03303-6.

## Introduction

Cancer is a well-established risk factor for venous thromboembolism (VTE) and a major contributor to morbidity and mortality [1, 2]. Cancer-associated thrombosis (CAT) represents a clinically significant complication, requiring accurate risk stratification to guide thromboprophylaxis. Current guidelines recommend pharmacological prophylaxis for ambulatory cancer patients classified as high risk according to validated risk assessment models (RAMs) [3–5].

Recent meta-analyses and validation studies indicate that, despite the availability of multiple RAMs, predictive performance remains variable. Most models rely exclusively on clinical variables, and the Khorana score remains the most widely used tool. However, it has limited sensitivity and fails to identify a substantial proportion of patients who subsequently develop VTE outside the high-risk category. Its performance also varies by cancer type, with poorer discrimination reported in lung cancer, lymphoma, gastrointestinal malignancies, and heterogeneous populations [6, 7].

To improve predictive accuracy while maintaining clinical applicability, several modifications of the Khorana score have been proposed. Incorporation of biomarkers—particularly D-dimer—can enhance risk discrimination and positive predictive value by identifying high-risk phenotypes not captured by clinical variables alone. However, comparative studies have not consistently demonstrated a clear superiority of modified models. Among the most widely studied are ONKOTEV, PROTECHT, CONKO, and Vienna CATS. Although ONKOTEV shows acceptable discrimination, overall performance across models remains modest, with no consistent superiority over the original Khorana score in pairwise or network meta-analyses [8–10].

Importantly, the performance of these models depends on the populations in which they are applied. Therefore, rigorous external validation of RAMs is crucial to determine their generalizability and to guide the refinement of risk stratification approaches and clinical guidelines. In Brazil, data on CAT incidence and real-world RAM performance are scarce, representing a significant gap in the literature.

Therefore, this study aimed to externally validate and compare the predictive performance and clinical utility of four established VTE risk assessment models (Khorana [11], PROTECHT [12], CONKO [13], and Vienna CATS [14]) in a large prospective cohort of ambulatory cancer patients from a Brazilian tertiary-care registry.

## Methods

### Study population

This study was designed as a prospective external validation of four established VTE RAMs in cancer patients: Khorana [11], PROTECHT [12], CONKO [13], and Vienna CATS [14]. The objective was to evaluate and compare their discriminative performance in an independent cohort.

This was a prospective, multicenter cohort study conducted at tertiary-care academic institutions in Campinas, São Paulo, Brazil. Participants were recruited from three affiliated public institutions: the Center for Thromboembolic Diseases (CDT/Hemocentro), the Women’s Comprehensive Health Care Center (CAISM), and the Clinical Hospital of the Faculty of Medical Sciences, all linked to the State University of Campinas (UNICAMP). It should be noted that, despite the multicenter design, all centers were located within a restricted geographic area in the state of São Paulo, southeastern Brazil, which may limit generalizability.

The study was approved by the local institutional review board (CAAE: 87212318.7.0000.5404) and conducted in accordance with the Declaration of Helsinki. All participants provided written informed consent. Data were prospectively collected and managed using REDCap (Research Electronic Data Capture).

The study followed the Transparent Reporting of a Multivariable Prediction Model for Individual Prognosis or Diagnosis (TRIPOD) statement [15], with the completed checklist provided in Table S2 (Supplementary Material).

Between July 2016 and July 2020, consecutive ambulatory adult patients (≥ 18 years) with histologically confirmed solid or hematologic malignancies were enrolled prior to initiation of a new chemotherapy regimen. Eligible patients had a newly diagnosed cancer or disease progression after complete remission. Tumor types included brain, breast, lung, stomach, lower gastrointestinal tract, pancreas, kidney, prostate, gynecologic malignancies, sarcomas, multiple myeloma, and lymphoma.

Exclusion criteria were objectively confirmed venous or arterial thromboembolism within the previous three months and ongoing therapeutic anticoagulation for any indication. Concomitant antiplatelet therapy was permitted. A small number of patients (< 20%) received thromboprophylaxis, including aspirin, clopidogrel, enoxaparin, rivaroxaban, and unfractionated heparin. Patients not scheduled to receive systemic chemotherapy (e.g., radiotherapy, hormonal therapy, surgery alone, or no active treatment) were excluded.

At baseline, a structured clinical assessment was performed, including tumor site and histology. Patients received standardized education on signs and symptoms of VTE and were instructed to report suspected events promptly. Peripheral blood samples were collected at study entry for measurement of D-dimer and soluble p-selectin.

Participants were followed for up to 12 months or until VTE occurrence, death, loss to follow-up, or withdrawal of consent, whichever occurred first. A monthly telephone follow-up was conducted to assess VTE events and treatment status. Medical records were systematically reviewed, and in cases of non-response, relatives or institutional records were consulted.

### Outcome definition and adjudication

The primary outcome was objectively confirmed symptomatic or fatal VTE within 12 months of study entry, including deep vein thrombosis (DVT) and pulmonary embolism (PE).

Routine screening for asymptomatic VTE was not performed. Diagnostic imaging was conducted only in patients with clinical suspicion. DVT was confirmed by duplex ultrasonography, and PE by computed tomography pulmonary angiography or ventilation–perfusion scintigraphy.

For patients who died during follow-up, death certificates and autopsy reports were reviewed to identify fatal PE. Outcomes were determined based on imaging and clinical documentation generated during routine care.

In this study, no formal blinding to baseline predictors was implemented.

### Risk score assessment

All variables required to calculate the Khorana, PROTECHT, CONKO, and Vienna CATS scores were collected at baseline, prior to chemotherapy initiation. Risk scores were calculated strictly according to their original published definitions, without modification or recalibration. Predictors were not transformed, reweighted, or re-estimated.

High-risk classification for Khorana, PROTECHT, and CONKO scores was defined using the conventional ≥ 3-point threshold, with a prespecified ≥ 2-point threshold evaluated in sensitivity analyses. For Vienna CATS, conventional (≥ 5 points) and alternative (≥ 4 points) thresholds were assessed.

### Statistical analyses

Baseline characteristics were summarized for the overall cohort and stratified by VTE status. Continuous variables were reported as medians with interquartile ranges (IQR) and compared using the Mann–Whitney U test. Categorical variables were expressed as counts and percentages and compared using Fisher’s exact test. All tests were two-sided, with *P* < 0.05 considered statistically significant.

Discriminative performance was assessed using receiver operating characteristic (ROC) curve analysis, with calculation of the area under the curve (AUC) for the 12-month VTE outcome. Sensitivity, specificity, positive predictive value (PPV), and negative predictive value (NPV) were calculated at predefined thresholds and across all observed score values.

95% confidence intervals (CI) were estimated using nonparametric bootstrap resampling (10,000 iterations). Pairwise comparisons of AUCs were performed using DeLong’s test for correlated ROC curves.

Time-to-event analyses used the study entry (baseline assessment prior to chemotherapy) as the time origin. Patients were censored at the last follow-up or 12 months. Cumulative incidence curves were generated to visualize VTE risk over time by risk category.

To explore interactions among score components, UpSet plots were constructed for each model, combining binary indicators of individual components with their respective weights to identify frequent feature intersections associated with VTE.

As this was a pure external validation study, no model updating, recalibration, or coefficient re-estimation was performed. The sample size was determined by the available cohort, with no formal calculation. Formal calibration analyses were not performed because the models provide categorical risk classifications rather than continuous probabilities. However, observed vs. expected event rates were compared descriptively. No missing baseline data were present; therefore, imputation was not required.

All analyses were performed using MATLAB (version R2025a; MathWorks, Natick, MA, USA).

## Results

### Population characteristics

**Fig. 1:**
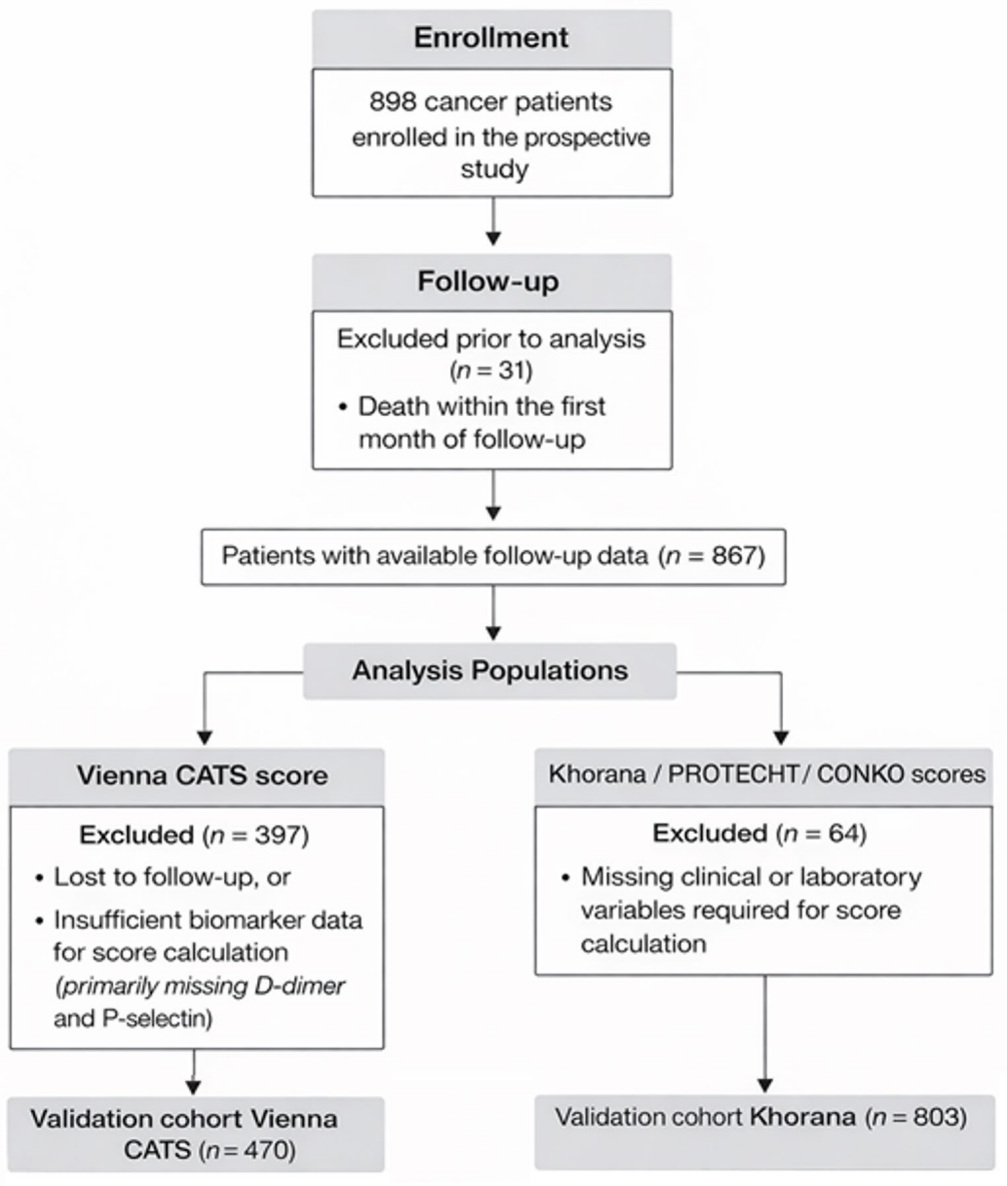
Flowchart of the study population

Of 898 enrolled patients, 867 were evaluable after excluding 31 who died within the first month (none with documented VTE; all deaths attributed to cancer progression) (Fig. 1). The Khorana, PROTECHT, and CONKO scores were validated in 803 patients with complete data, while Vienna CATS was evaluated in a predefined biomarker subcohort of 470 patients. Accordingly, comparisons involving Vienna CATS were restricted to this subcohort.

During follow-up, 36 of 803 patients (4.5%) developed VTE. Baseline characteristics are summarized in Table 1. Baseline demographic variables were largely comparable between patients with and without VTE. Age did not differ significantly (median 65.0 vs. 63.0 years, *p* = 0.189), nor did sex distribution (52.8% vs. 50.8% female, *p* = 0.866) or body mass index (BMI) (median 23.5 vs. 25.0 kg/m², *p* = 0.744). Statin use was more frequent among patients who developed VTE (30.6% vs. 16.8%, *p* = 0.043). A prior history of thrombosis was also significantly more common in the VTE group (19.4% vs. 5.9%, *p* = 0.006), supporting its established role as a risk factor. Other clinical variables, including antidiabetic medication use, hormone therapy exposure, recent surgery, immobilization, functional status, education level, lifestyle factors, and chemotherapy regimen, did not differ significantly between groups.

Marked differences were observed in tumor histology and site. Adenocarcinoma was significantly more prevalent among patients who developed VTE (61.1% vs. 38.1%, *p* = 0.008). Glioma was also more frequent in the VTE group (8.3% vs. 1.6%, *p* = 0.026). Regarding tumor location, pancreatic cancer showed a strong association with VTE (13.9% vs. 3.4%, *p* = 0.010). Central nervous system tumors were more common among VTE patients (8.3% vs. 1.7%, *p* = 0.031), and cancers of the ureters/renal pelvis were observed exclusively in the VTE group (2.8% vs. 0.0%, *p* = 0.045), although absolute numbers were small. Conversely, lymphomas were less frequent among patients who developed VTE (0.0% vs. 11.1%, *p* = 0.025).

Baseline laboratory findings were broadly similar between groups, with one notable exception. D-dimer levels were markedly higher in patients who developed VTE (median 1,852.5 vs. 700.0 ng/mL Fibrinogen Equivalent Units (FEU), *p* < 0.001), consistent with increased thrombotic activity. In contrast, soluble P-selectin concentrations did not differ significantly (*p* = 0.488). Hemoglobin, hematocrit, platelet count, total white blood cell count, absolute neutrophil count, red blood cell count, and red cell distribution width showed no statistically significant differences between groups.

Regarding cancer treatment, it is important to describe the therapeutic modalities administered explicitly. In the overall cohort (*n* = 803), 77.95% of patients received cytotoxic chemotherapy, 7.47% received cytotoxic chemotherapy combined with endocrine therapy, and 9.96% received cytotoxic chemotherapy combined with immunobiologic therapy. Smaller proportions of patients received cytotoxic chemotherapy in combination with immunomodulatory drugs (2.24%), while only 2.36% received immunomodulatory drugs alone.

The presence of metastatic disease among tumors was also included in the analysis. Although metastatic disease was numerically more frequent in patients who developed VTE (8.3% vs. 3.9%), this difference did not reach statistical significance (*p* = 0.180).

Pharmacological thromboprophylaxis during follow-up was additionally assessed. Most patients did not receive prophylaxis (82.6%), while enoxaparin was the most frequently used agent (11.5%), followed by aspirin (3.0%) and rivaroxaban (1.1%). The use of thromboprophylaxis did not differ significantly between patients with and without VTE, although a numerically higher proportion of patients on rivaroxaban was observed in the VTE group (5.6% vs. 0.9%, *p* = 0.058). Overall, these findings suggest that neither treatment modality, metastatic status, nor thromboprophylaxis use significantly differed between groups in this cohort.

Table 2 summarizes baseline demographic, clinical, tumor-related, and laboratory characteristics of the 470 patients included in the Vienna CATS validation cohort, among whom 24 (5.1%) developed objectively confirmed VTE during follow-up. Baseline demographic characteristics were comparable between groups. Age was numerically higher in the VTE group but did not reach statistical significance (median 66.5 vs. 62.0 years, *p* = 0.082). Sex distribution (54.2% vs. 50.2% female, *p* = 0.835) and BMI (median 24.9 vs. 25.2 kg/m², *p* = 0.712) were similar between groups. A prior history of thrombosis was significantly more frequent among patients who developed VTE (29.2% vs. 5.2%, *p* < 0.001), reinforcing its established role as an important risk factor. Other variables did not differ significantly between groups.

Adenocarcinoma histology was significantly more prevalent among patients who developed VTE (66.7% vs. 39.0%, *p* = 0.010). With respect to tumor location, colon cancer was more frequent in the VTE group (20.8% vs. 7.0%, *p* = 0.029). No statistically significant differences were observed for pancreatic cancer (12.5% vs. 4.0%, *p* = 0.085) or other tumor sites, although small event numbers limited several comparisons.

D-dimer levels were significantly higher in patients who developed VTE (median 1,696.5 vs. 738.0 ng/mL FEU, *p* = 0.004), consistent with increased thrombotic activity. In contrast, soluble P-selectin concentrations did not differ significantly between groups (*p* = 0.355). No other hematologic variable showed statistically significant differences.

For the cohort (*n* = 470), the distribution of cancer treatments was as follows: 80.85% of patients received cytotoxic chemotherapy, 6.80% received cytotoxic chemotherapy combined with endocrine therapy, and 8.9% received cytotoxic chemotherapy combined with immunobiologic therapy. Smaller proportions received cytotoxic chemotherapy in combination with immunomodulatory drugs (2.12%), while 1.27% received immunomodulatory drugs alone. No statistically significant differences were observed between groups.

Metastatic disease among tumors was also evaluated and presented as not statistically significant (*p* = 0.194). Regarding pharmacological thromboprophylaxis during follow-up, enoxaparin was the most commonly used agent, followed by aspirin and rivaroxaban. No significant differences were observed between groups in the use of thromboprophylaxis.

To further assess potential selection bias related to biomarker availability, we compared baseline characteristics between patients with available D-dimer and soluble P-selectin measurements (*n* = 470) and those without these data (*n* = 333) (Table S1, Supplementary Material). Overall, the two groups were broadly comparable across most demographic, clinical, and laboratory variables. Importantly, the incidence of VTE did not differ significantly between groups (5.1% vs. 3.6%, *p* = 0.387).

However, several statistically significant differences were observed. Patients without biomarker data had a higher proportion of individuals with no formal education (14.7% vs. 7.4%, *p* < 0.001), whereas those with available biomarkers were more likely to have secondary education (28.9% vs. 22.5%, *p* = 0.042). Differences were also noted in smoking status, with a higher proportion of former smokers in the biomarker-unavailable group (40.2% vs. 33.0%, *p* = 0.037). Regarding tumor characteristics, carcinoma histology was more frequent among patients without biomarker data (41.1% vs. 28.9%, *p* < 0.001), whereas multiple myeloma was more common in those with available biomarkers (6.6% vs. 2.7%, *p* = 0.013). Differences were also observed in tumor location, including breast cancer (17.4% vs. 10.9%, *p* = 0.009), head and neck tumors (6.6% vs. 2.6%, *p* = 0.007), and bone marrow involvement (2.7% vs. 6.6%, *p* = 0.013).

Taken together, these findings suggest that biomarker availability was not completely random, indicating the potential for selection bias. Nevertheless, given the overall similarity across most variables and the absence of a significant difference in VTE incidence, the biomarker-defined subcohort appears reasonably representative of the full study population.

Accordingly, comparisons between risk models were performed within the same populations to minimize bias: the Khorana, PROTECHT, and CONKO scores were evaluated in the full cohort (*n* = 803), while comparisons involving the Vienna CATS model were restricted to the biomarker-available subcohort (*n* = 470). Direct comparisons across different populations should therefore be interpreted with caution.

**Table 1:** Baseline demographic, clinical, tumor-related, and laboratory characteristics of patients evaluated using the Khorana, PROTECHT, and CONKO risk scores, stratified by venous thromboembolism status

Characteristic	All patients (*N* = 803)	No VTE (*n* = 767)	VTE^*^ (*n* = 36)	*P* †
**Age**,** years**,** median (25th-75th percentile)**	63.0 (53.0–71.0)	63.0 (53.0–71.0)	65.0 (56.5–75.5)	0.189
**Female sex**,** % (n)**	50.9% (409)	50.8% (390)	52.8% (19)	0.866
**Body mass index**,** kg/m**^**2**^, **median (25th-75th percentile)**	24.9 (21.6–28.8)	25.0 (21.6–28.9)	23.5 (22.1–28.4)	0.744
**Statin use**,** % (n)**	17.4% (140)	16.8% (129)	30.6% (11)	**0.043**
**Antidiabetic medication**,** % (n)**	15.8% (127)	15.4% (118)	25.0% (9)	0.156
**Hormone therapy use**,** % (n)**				
Current use (oral contraceptives, injectable contraceptives, or hormone replacement therapy)	27.9% (224)	28.4% (218)	16.7% (6)	0.181
No use	23.4% (188)	22.8% (175)	36.1% (13)	0.072
**Previous thrombosis**,** % (n)**	6.5% (52)	5.9% (45)	19.4% (7)	**0.006**
**Surgery > 30 min with anesthesia**,** % (n)**	71.7% (576)	72.0% (552)	66.7% (24)	0.570
**Any immobilization**,** % (n)**	18.9% (152)	18.9% (145)	19.4% (7)	1.000
**World Health Organization functional status ≥ 2**,** % (n)**	2.2% (18)	2.2% (17)	2.8% (1)	0.566
**Education**,** % (n)**				
No formal education	10.6% (85)	10.6% (81)	11.1% (4)	0.786
Primary education	53.7% (431)	53.8% (413)	50.0% (18)	0.733
Secondary education	26.3% (211)	26.3% (202)	25.0% (9)	1.000
Higher education	9.5% (76)	9.3% (71)	13.9% (5)	0.375
**Lifestyle factors**,** % (n)**				
Current smoker	11.1% (89)	11.2% (86)	8.3% (3)	0.788
Former smoker	36.0% (289)	35.5% (272)	47.2% (17)	0.158
Never smoker	52.9% (425)	53.3% (409)	44.4% (16)	0.311
Current alcohol use	3.7% (30)	3.8% (29)	2.8% (1)	1.000
Former alcohol use	13.0% (104)	13.0% (100)	11.1% (4)	1.000
Never alcohol use	83.3% (669)	83.2% (638)	86.1% (31)	0.820
**Chemotherapy regimen**,** % (n)**				
Platinum-based chemotherapy	15.8% (127)	15.6% (120)	19.4% (7)	0.489
Gemcitabine-based chemotherapy	1.2% (10)	1.3% (10)	0.0% (0)	1.000
Platinum plus gemcitabine chemotherapy	7.6% (61)	7.4% (57)	11.1% (4)	0.344
**Tumor histology**,** % (n)**				
Adenocarcinoma	39.1% (314)	38.1% (292)	61.1% (22)	**0.008**
Carcinoma	34.0% (273)	34.3% (263)	27.8% (10)	0.476
Germ cell tumor	0.2% (2)	0.3% (2)	0.0% (0)	1.000
Glioma	1.9% (15)	1.6% (12)	8.3% (3)	**0.026**
Hodgkin lymphoma	2.5% (20)	2.6% (20)	0.0% (0)	1.000
Non-Hodgkin lymphoma	8.2% (66)	8.6% (66)	0.0% (0)	0.065
Melanoma	1.1% (9)	1.2% (9)	0.0% (0)	1.000
Multiple myeloma	5.0% (40)	5.1% (39)	2.8% (1)	1.000
Trophoblastic neoplasia	0.1% (1)	0.1% (1)	0.0% (0)	1.000
Sarcoma	2.4% (19)	2.5% (19)	0.0% (0)	1.000
Neuroendocrine tumor	0.7% (6)	0.8% (6)	0.0% (0)	1.000
Other histologies	4.7% (38)	5.0% (38)	0.0% (0)	0.407
**Metastatic**,** % (n)**				
Yes	4.1% (33)	3.9% (30)	8.3% (3)	0.180
No	80.0% (642)	79.5% (610)	88.9% (32)	0.205
Not reported	15.9% (128)	16.6% (127)	2.8% (1)	**0.020**
**Tumor location**,** % (n)**				
Pancreas	3.9% (31)	3.4% (26)	13.9% (5)	**0.010**
Stomach	8.7% (70)	8.5% (65)	13.9% (5)	0.232
Lung / Pleura	6.1% (49)	6.0% (46)	8.3% (3)	0.477
Ovarian	0.7% (6)	0.7% (5)	2.8% (1)	0.241
Uterine (corpus)	0.6% (5)	0.7% (5)	0.0% (0)	1.000
Cervical	1.6% (13)	1.7% (13)	0.0% (0)	1.000
Testicular	1.0% (8)	0.9% (7)	2.8% (1)	0.308
Lymph nodes and spleen	10.6% (85)	11.1% (85)	0.0% (0)	**0.025**
Renal	2.5% (20)	2.6% (20)	0.0% (0)	1.000
Bladder	1.7% (14)	1.8% (14)	0.0% (0)	1.000
Breast	13.7% (110)	14.1% (108)	5.6% (2)	0.212
Colon	6.5% (52)	6.1% (47)	13.9% (5)	0.076
Rectum	9.5% (76)	9.5% (73)	8.3% (3)	1.000
Esophagus	5.7% (46)	5.7% (44)	5.6% (2)	1.000
Liver	2.9% (23)	2.6% (20)	8.3% (3)	0.079
Small intestine	2.7% (22)	2.7% (21)	2.8% (1)	1.000
Bone and cartilage	2.7% (22)	2.9% (22)	0.0% (0)	0.618
Soft tissue	0.2% (2)	0.3% (2)	0.0% (0)	1.000
Skin	1.2% (10)	1.3% (10)	0.0% (0)	1.000
Central nervous system	2.0% (16)	1.7% (13)	8.3% (3)	**0.031**
Head and neck	4.2% (34)	4.4% (34)	0.0% (0)	0.395
Bone marrow (myeloma)	5.0% (40)	5.1% (39)	2.8% (1)	1.000
Mediastinum	0.1% (1)	0.1% (1)	0.0% (0)	1.000
Peritoneum	0.5% (4)	0.5% (4)	0.0% (0)	1.000
Prostate	2.9% (23)	3.0% (23)	0.0% (0)	0.619
Adrenal	0.5% (4)	0.5% (4)	0.0% (0)	1.000
Biliary tract	1.0% (8)	1.0% (8)	0.0% (0)	1.000
Anal canal	0.4% (3)	0.4% (3)	0.0% (0)	1.000
Penis	0.2% (2)	0.3% (2)	0.0% (0)	1.000
Thymus / lymph node / spleen	0.4% (3)	0.4% (3)	0.0% (0)	1.000
Ureters / renal pelvis	0.1% (1)	0.0% (0)	2.8% (1)	**0.045**
**Laboratory parameters**,** median (25th–75th percentile)**				
Platelet count (×10⁹/L)	271.0 (210.0–346.8)	269.0 (210.0–346.8)	279.0 (225.5–348.0)	0.506
Hemoglobin (g/dL)	12.5 (10.8–13.7)	12.5 (10.9–13.7)	11.8 (9.3–13.9)	0.160
Total white blood cell count (×10⁹/L)	7.7 (5.9–9.6)	7.6 (5.9–9.5)	8.0 (6.6–10.7)	0.135
Red blood cell count (×10¹²/L)	4.3 (3.8–4.7)	4.3 (3.8–4.7)	4.0 (3.4–4.7)	0.176
D-dimer (ng/mL FEU) ‡	715.0 (367.0–1429.0)	700.0 (362.0–1318.0)	1852.5 (685.0–3070.0)	**< 0.001**
Soluble p-selectin (ng/mL) ‡	90.3 (58.7–139.2)	90.3 (58.5–138.0)	100.6 (63.4–180.3)	0.488
Hematocrit (%)	38.0 (33.4–41.5)	38.0 (33.5–41.5)	35.9 (28.8–41.5)	0.092
Absolute neutrophil count (×10⁹/L)	4.7 (3.5–6.4)	4.6 (3.5–6.4)	5.5 (3.5–7.3)	0.126
Red cell distribution width (%)	14.1 (13.3–15.4)	14.1 (13.3–15.4)	14.1 (13.1–16.4)	0.706
**Antiplatelet and Anticoagulant prophylaxis**,** n (%)**				
Aspirin	3.0% (24)	3.1% (24)	0.0% (0)	0.620
Clopidogrel	0.2% (2)	0.3% (2)	0.0% (0)	1.000
Enoxaparin	11.5% (92)	11.6% (89)	8.3% (3)	0.789
Rivaroxaban	1.1% (9)	0.9% (7)	5.6% (2)	0.058
Unfractionated heparin	1.6% (13)	1.7% (13)	0.0% (0)	1.000
No pharmacologic antiplatelet and anticoagulation	82.6% (663)	82.4% (632)	86.1% (31)	0.822

*VTE includes objectively confirmed venous thromboembolism events during follow-up

†P values were calculated using the Mann–Whitney U test for continuous variables and Fisher’s exact test for categorical variables. A two-sided *P* < 0.05 was considered statistically significant

‡ Values were calculated among patients with available measurements for D-dimer (*n* = 554; 26 with VTE) and soluble p-selectin (*n* = 576; 28 with VTE)

**Table 2:** Baseline demographic, clinical, tumor-related, and laboratory characteristics of patients included in the evaluation of the Vienna CATS risk score, stratified by venous thromboembolism status

Characteristic	All patients (*N* = 470)	No VTE (*n* = 446)	VTE^*^ (*n* = 24)	*P* †
**Age**,** years**,** median (25th-75th percentile)**	62.5 (53.0–71.0)	62.0 (53.0–71.0)	66.5 (58.0–77.0)	0.082
**Female sex**,** % (n)**	50.4% (237)	50.2% (224)	54.2% (13)	0.835
**Body mass index**,** kg/m**^**2**^, **median (25th-75th percentile)**	25.1 (22.0–28.9)	25.2 (21.9–28.9)	24.9 (22.7–28.8)	0.712
**Statin use**,** % (n)**	16.8% (79)	16.1% (72)	29.2% (7)	0.155
**Antidiabetic medication**,** % (n)**	16.0% (75)	15.2% (68)	29.2% (7)	0.084
**Hormone therapy use**,** % (n)**				
Current use (oral contraceptives, injectable contraceptives, or hormone replacement therapy)	24.3% (114)	24.7% (110)	16.7% (4)	0.470
No use	26.2% (123)	25.6% (114)	37.5% (9)	0.232
**Previous thrombosis**,** % (n)**	6.4% (30)	5.2% (23)	29.2% (7)	**< 0.001**
**Surgery > 30 min with anesthesia**,** % (n)**	69.1% (325)	70.0% (312)	54.2% (13)	0.115
**Any immobilization**,** % (n)**	17.2% (81)	17.3% (77)	16.7% (4)	1.000
**World Health Organization functional status ≥ 2**,** % (n)**	2.3% (11/469)	2.2% (10/445)	4.2% (1)	0.442
**Education**,** % (n)**				
No formal education	7.4% (35)	7.4% (33)	8.3% (2)	0.697
Primary education	54.5% (256)	54.7% (244)	50.0% (12)	0.679
Secondary education	28.9% (136)	29.1% (130)	25.0% (6)	0.819
Higher education	9.1% (43)	8.7% (39)	16.7% (4)	0.261
**Lifestyle factors**,** % (n)**				
Current smoker	11.7% (55)	11.7% (52)	12.5% (3)	0.752
Former smoker	33.0% (155)	32.3% (144)	45.8% (11)	0.185
Never smoker	55.3% (260)	56.1% (250)	41.7% (10)	0.207
Current alcohol use	4.3% (20)	4.3% (19)	4.2% (1)	1.000
Former alcohol use	12.3% (58)	12.6% (56)	8.3% (2)	0.754
Never alcohol use	83.4% (392)	83.2% (371)	87.5% (21)	0.780
**Chemotherapy regimen % (n)**				
Platinum-based chemotherapy	15.5% (73)	15.2% (68)	20.8% (5)	0.399
Gemcitabine-based chemotherapy	1.5% (7)	1.6% (7)	0.0% (0)	1.000
Platinum plus gemcitabine chemotherapy	7.0% (33)	6.7% (30)	12.5% (3)	0.233
**Tumor histology**,** % (n)**				
Adenocarcinoma	40.4% (190)	39.0% (174)	66.7% (16)	**0.010**
Carcinoma	28.9% (136)	29.4% (131)	20.8% (5)	0.490
Germ cell tumor	0.0% (0)	0.0% (0)	0.0% (0)	1.000
Glioma	2.6% (12)	2.2% (10)	8.3% (2)	0.121
Hodgkin lymphoma	2.8% (13)	2.9% (13)	0.0% (0)	1.000
Non-Hodgkin lymphoma	9.8% (46)	10.3% (46)	0.0% (0)	0.153
Melanoma	0.6% (3)	0.7% (3)	0.0% (0)	1.000
Multiple myeloma	6.6% (31)	6.7% (30)	4.2% (1)	1.000
Trophoblastic neoplasia	0.0% (0)	0.0% (0)	0.0% (0)	1.000
Sarcoma	3.2% (15)	3.4% (15)	0.0% (0)	1.000
Neuroendocrine tumor	0.6% (3)	0.7% (3)	0.0% (0)	1.000
Other histologies	4.5% (21)	4.7% (21)	0.0% (0)	0.616
**Metastatic**,** % (n)**				
Yes	3.4% (16)	3.1% (14)	8.3% (2)	0.194
No	77.4% (364)	76.9% (343)	87.5% (21)	0.317
Not reported	19.1% (90)	20.0% (89)	4.2% (1)	0.062
**Tumor location**,** % (n)**				
Pancreas	4.5% (21)	4.0% (18)	12.5% (3)	0.085
Stomach	8.7% (41)	8.3% (37)	16.7% (4)	0.147
Lung / Pleura	4.7% (22)	4.3% (19)	12.5% (3)	0.095
Ovarian	1.1% (5)	0.9% (4)	4.2% (1)	0.231
Uterine (corpus)	0.4% (2)	0.4% (2)	0.0% (0)	1.000
Cervical	1.9% (9)	2.0% (9)	0.0% (0)	1.000
Testicular	0.6% (3)	0.7% (3)	0.0% (0)	1.000
Lymph nodes and spleen	12.3% (58)	13.0% (58)	0.0% (0)	0.058
Renal	1.9% (9)	2.0% (9)	0.0% (0)	1.000
Bladder	1.7% (8)	1.8% (8)	0.0% (0)	1.000
Breast	10.9% (51)	11.4% (51)	0.0% (0)	0.094
Colon	7.7% (36)	7.0% (31)	20.8% (5)	**0.029**
Rectum	11.1% (52)	11.0% (49)	12.5% (3)	0.740
Esophagus	5.1% (24)	5.4% (24)	0.0% (0)	0.625
Liver	2.6% (12)	2.2% (10)	8.3% (2)	0.121
Small intestine	2.3% (11)	2.5% (11)	0.0% (0)	1.000
Bone and cartilage	3.6% (17)	3.8% (17)	0.0% (0)	1.000
Soft tissue	0.4% (2)	0.4% (2)	0.0% (0)	1.000
Skin	0.9% (4)	0.9% (4)	0.0% (0)	1.000
Central nervous system	2.8% (13)	2.5% (11)	8.3% (2)	0.138
Head and neck	2.6% (12)	2.7% (12)	0.0% (0)	1.000
Bone marrow (myeloma)	3.6% (17)	3.8% (17)	0.0% (0)	1.000
Mediastinum	0.0% (0)	0.0% (0)	0.0% (0)	1.000
Peritoneum	0.4% (2)	0.4% (2)	0.0% (0)	1.000
Prostate	3.2% (15)	3.4% (15)	0.0% (0)	1.000
Adrenal	0.2% (1)	0.2% (1)	0.0% (0)	1.000
Biliary tract	1.1% (5)	1.1% (5)	0.0% (0)	1.000
Anal canal	0.4% (2)	0.4% (2)	0.0% (0)	1.000
Penis	0.2% (1)	0.2% (1)	0.0% (0)	1.000
Thymus / lymph node / spleen	0.2% (1)	0.2% (1)	0.0% (0)	1.000
Ureters / renal pelvis	0.0% (0)	0.0% (0)	0.0% (0)	1.000
**Laboratory parameters**,** median (25th–75th percentile)**				
Platelet count (×10⁹/L)	267.0 (205.0–349.0)	263.5 (204.0–349.0)	279.0 (250.5–348.0)	0.313
Hemoglobin (g/dL)	12.4 (10.8–13.7)	12.4 (10.9–13.7)	11.8 (9.2–13.9)	0.210
Total white blood cell count (×10⁹/L)	7.7 (6.1–9.6)	7.7 (6.1–9.3)	8.7 (6.4–11.6)	0.130
Red blood cell count (×10¹²/L)	4.3 (3.7–4.8)	4.3 (3.7–4.8)	4.0 (3.4–4.7)	0.414
D-dimer (ng/mL FEU)	745.0 (374.0–1551.0)	738.0 (371.0–1429.0)	1696.5 (660.0–2822.0)	**0.004**
Soluble p-selectin (ng/mL)	93.7 (61.4–147.9)	93.6 (61.0–147.8)	116.0 (69.2–180.3)	0.355
Hematocrit (%)	37.8 (33.2–41.7)	38.0 (33.5–41.7)	35.3 (28.8–41.8)	0.162
Absolute neutrophil count (×10⁹/L)	4.7 (3.4–6.4)	4.6 (3.4–6.3)	5.5 (3.4–8.0)	0.251
Red cell distribution width (%)	14.0 (13.2–15.3)	13.9 (13.2–15.3)	14.1 (13.1–16.4)	0.618
**Antiplatelet and Anticoagulant prophylaxis**,** n (%)**				
Aspirin	4.0% (19)	4.3% (19)	0.0% (0)	0.614
Clopidogrel	0.2% (1)	0.2% (1)	0.0% (0)	1.000
Enoxaparin	12.6% (59)	12.8% (57)	8.3% (2)	0.754
Rivaroxaban	1.1% (5)	0.9% (4)	4.2% (1)	0.231
Unfractionated heparin	1.3% (6)	1.3% (6)	0.0% (0)	1.000
No pharmacologic antiplatelet and anticoagulation	80.9% (380)	80.5% (359)	87.5% (21)	0.594

*VTE includes objectively confirmed venous thromboembolism events during follow-up

†P values were calculated using the Mann–Whitney U test for continuous variables and Fisher’s exact test for categorical variables. A two-sided *P* < 0.05 was considered statistically significant

### Follow-up and outcomes

Among the 803 patients included in the primary analysis, 36 (4.5%) developed symptomatic, objectively confirmed VTE during follow-up. The median time to VTE occurrence was 75 days (95% CI, 39–115). Of the 36 events, 15 (41.7%) were PE, and 15 (41.7%) were DVT of the lower extremities. Three events (8.3%) corresponded to splanchnic vein thrombosis involving the portal vein, and three events (8.3%) were upper-extremity DVT.

### Performance of Khorana, PROTECHT, and CONKO scores

The performance results of Khorana, PROTECHT, and CONKO are presented in Table 3. The results demonstrated comparable but limited predictive utility for VTE at their conventional high-risk thresholds. At the standard cutoff of ≥ 3, the Khorana score showed a sensitivity of 30.6% (95% CI 16.1–46.4) and a specificity of 86.4% (95% CI 84.0–88.9). This indicates that while the score presents a high NPV of 96.4%, it fails to capture the majority of patients who ultimately develop thrombosis. Similarly, the PROTECHT score at the ≥ 3 threshold had a sensitivity of 33.3% (95% CI 18.8–50.0) and a specificity of 84.0% (95% CI 81.3–86.6), with a low PPV of 8.9%, suggesting modest improvement over Khorana despite incorporating chemotherapy-related risk factors. The CONKO score performed similarly, yielding a sensitivity of 30.6% (95% CI 16.1–46.4) and a specificity of 86.6% (95% CI 84.2–89.0). These findings highlight a shared structural limitation: all three scores fail to identify approximately 65–70% of thrombotic events, limiting their utility for targeting thromboprophylaxis.

Exploring alternative point thresholds revealed the inherent trade-off between sensitivity and precision. Lowering the cutoff to ≥ 1 increased sensitivity to 63.9–69.4%, but specificity dropped below 40% across all models, undermining the ability to discriminate high-risk patients. Conversely, very high cutoffs (≥ 4 or ≥ 5) achieved high specificity (> 96%) and a modest increase in PPV (up to 33.3% for CONKO ≥ 5), yet sensitivity fell below 9%, capturing only a small fraction of total events. This pattern demonstrates that extreme point scores enrich for risk but fail to identify the majority of patients experiencing VTE.

**Fig. 2:**
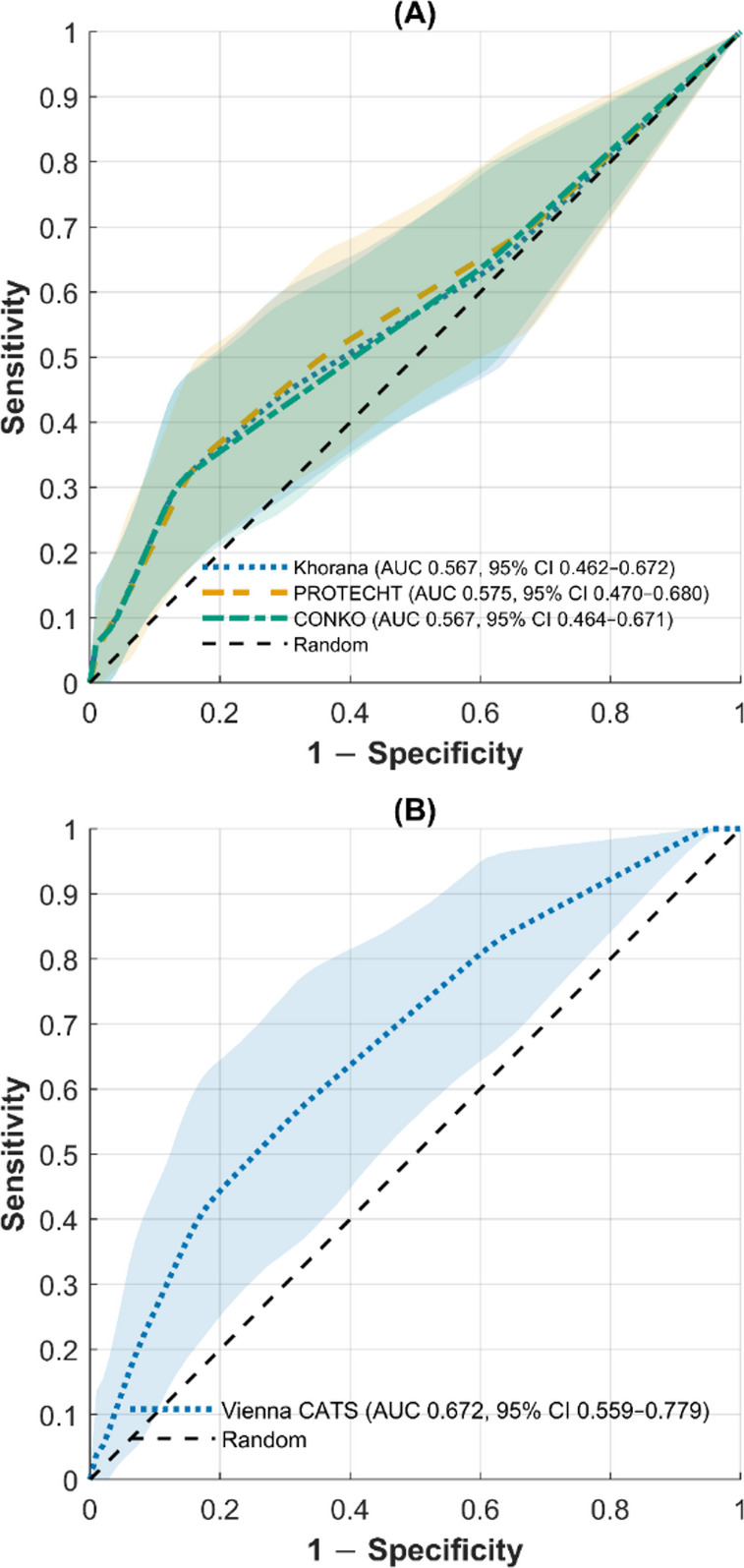
Receiver operating characteristic curves with 95% confidence intervals for the (**A**) Khorana, PROTECHT, and CONKO scores, and (**B**) the Vienna CATS score

The overall discriminative performance of Khorana, PROTECHT, and CONKO was limited and highly comparable, as reflected in overlapping ROC curves (Fig. 2A). AUC values were modest: 0.567 (95% CI 0.462–0.672) for Khorana, 0.575 (95% CI 0.470–0.680) for PROTECHT, and 0.567 (95% CI 0.464–0.671) for CONKO. Notably, all 95% CIs included the reference threshold (AUC = 0.500, y = x line), indicating that predictive performance was limited and only modestly above chance.

Statistical comparison of discriminative performance further confirmed the lack of superiority between these models. Pairwise AUC analysis using DeLong’s test showed no significant differences in AUC between the Khorana (0.567), PROTECHT (0.575), and CONKO (0.567) scores. Specifically, no statistically significant differences were observed between Khorana and PROTECHT (ΔAUC = − 0.008; z = − 0.52; *p* = 0.604), Khorana and CONKO (ΔAUC = 0.000; z = − 0.01; *p* = 0.992), or PROTECHT and CONKO (ΔAUC = 0.008; z = 0.45; *p* = 0.655). These findings indicate that the additional clinical variables incorporated into the PROTECHT and CONKO modifications do not meaningfully improve the discriminative performance of the original Khorana score for predicting one-year VTE risk in this cohort.

**Table 3:** Performance of the Khorana, PROTECHT, CONKO, and Vienna CATS risk scores for prediction of venous thromboembolism across predefined cutoff values

Score	Cutoff	Patients with exact score, % of cohort (*n*)	VTE events, % of all VTE (*n*)	Sensitivity % (95% CI)	Specificity % (95% CI)	PPV % (95% CI)	NPV % (95% CI)
Khorana	0	37.5 (301)	36.1 (13)	100.0 (100.0–100.0)	0.0 (0.0–0.0)	4.5 (3.1–6.0)	0.0 (0.0–0.0)
	1	31.9 (256)	19.4 (7)	63.9 (47.7–79.6)	37.6 (34.2–41.1)	4.6 (2.8–6.5)	95.7 (93.3–97.8)
	2	16.3 (131)	13.9 (5)	44.4 (28.6–60.7)	70.0 (66.8–73.2)	6.5 (3.6–9.8)	96.4 (94.8–97.8)
	3†	10.7 (86)	22.2 (8)	30.6 (16.1–46.4)	86.4 (84.0–88.9)	9.6 (4.6–15.4)	96.4 (94.9–97.7)
	4	2.7 (22)	2.8 (1)	8.3 (0.0–18.6)	96.6 (95.3–97.8)	10.3 (0.0–23.1)	95.7 (94.3–97.1)
	5	0.9 (7)	5.6 (2)	5.6 (0.0–14.3)	99.3 (98.7–99.9)	28.6 (0.0–66.7)	95.7 (94.2–97.1)
PROTECHT	0	32.4 (260)	30.6 (11)	100.0 (100.0–100.0)	0.0 (0.0–0.0)	4.5 (3.1–6.0)	0.0 (0.0–0.0)
	1	31.1 (250)	19.4 (7)	69.4 (53.9–84.6)	32.5 (29.2–35.9)	4.6 (2.9–6.5)	95.8 (93.2–98.1)
	2	19.7 (158)	16.7 (6)	50.0 (33.3–66.7)	64.2 (60.8–67.5)	6.1 (3.6–9.1)	96.5 (94.8–98.0)
	3†	10.3 (83)	19.4 (7)	33.3 (18.8–50.0)	84.0 (81.3–86.6)	8.9 (4.5–14.1)	96.4 (94.5–97.8)
	4	4.9 (39)	8.3 (3)	13.9 (3.2–26.3)	93.9 (92.1–95.6)	9.6 (2.1–18.7)	95.9 (94.4–97.2)
	5	1.6 (13)	5.6 (2)	5.6 (0.0–14.3)	98.6 (97.6–99.3)	15.4 (0.0–38.5)	95.7 (94.2–97.1)
CONKO	0	39.1 (314)	36.1 (13)	100.0 (100.0–100.0)	0.0 (0.0–0.0)	4.5 (3.1–6.0)	0.0 (0.0–0.0)
	1	31.4 (252)	22.2 (8)	63.9 (47.7–79.6)	39.2 (35.8–42.8)	4.7 (2.9–6.7)	95.9 (93.6–97.9)
	2	15.3 (123)	11.1 (4)	41.7 (25.7–57.9)	71.1 (67.8–74.2)	6.3 (3.5–9.6)	96.3 (94.7–97.7)
	3†	10.5 (84)	22.2 (8)	30.6 (16.1–46.4)	86.6 (84.2–89.0)	9.6 (4.6–15.5)	96.4 (94.9–97.7)
	4	3.0 (24)	2.8 (1)	8.3 (0.0–18.6)	96.5 (95.1–97.7)	10.0 (0.0–22.2)	95.7 (94.3–97.1)
	5	0.6 (5)	2.8 (1)	5.6 (0.0–14.3)	99.5 (98.9–99.9)	33.3 (0.0–80.0)	95.7 (94.3–97.1)
	6	0.1 (1)	2.8 (1)	2.8 (0.0–9.4)	100.0 (100.0–100.0)	100.0 (0.0–100.0)	95.6 (94.1–97.0)
Vienna CATS ‡	0	5.1 (24)	0.0 (0)	100.0 (100.0–100.0)	0.0 (0.0–0.0)	5.1 (3.2–7.2)	0.0 (0.0–0.0)
	1	30.9 (145)	16.7 (4)	100.0 (100.0–100.0)	5.4 (3.4–7.6)	5.4 (3.4–7.6)	100.0 (100.0–100.0)
	2	29.1 (137)	25.0 (6)	83.3 (66.7–96.4)	37.0 (32.6–41.6)	6.6 (4.0–9.5)	97.6 (95.0–99.5)
	3	16.4 (77)	16.7 (4)	58.3 (37.5–78.3)	66.4 (61.9–70.7)	8.5 (4.5–12.3)	96.7 (94.5–98.6)
	4	10.2 (48)	20.8 (5)	41.7 (22.2–62.6)	82.7 (79.1–86.1)	11.5 (5.2–18.5)	96.3 (94.3–98.2)
	5†	6.4 (30)	16.7 (4)	20.8 (5.0–38.9)	92.4 (89.8–94.7)	12.8 (2.9–24.2)	95.6 (93.5–97.5)
	6	1.5 (7)	0.0 (0)	4.2 (0.0–14.3)	98.2 (96.9–99.3)	11.1 (0.0–37.5)	95.0 (92.9–97.0)
	7	0.4 (2)	4.2 (1)	4.2 (0.0–14.3)	99.8 (99.3–100.0)	50.0 (0.0–100.0)	95.1 (93.0–97.0)

Percentages in the “Patients with exact score” column are calculated relative to the entire cohort; percentages in the “VTE events” column are calculated relative to all observed VTE events

Performance metrics correspond to the indicated cutoff (points ≥ cutoff)

95% confidence intervals were estimated using bootstrap resampling (*n* = 10,000)

†Cutoff ≥ 3 corresponds to the conventional high-risk definition of the Khorana, PROTECHT, and CONKO scores. A cutoff of ≥ 5 corresponds to the conventional high-risk definition of the Vienna CATS score

‡Performance of Vienna CATS was assessed on the cohort of 470 patients with available D-dimer and p-selectin values

### Performance of Vienna CATS score

The Vienna CATS score was evaluated in a sub-cohort of 470 patients with available D-dimer and P-selectin measurements, demonstrating a distinct profile characterized by high specificity at upper score thresholds. At the conventional high-risk cutoff of ≥ 5, the score achieved a specificity of 92.4% (95% CI 89.8–94.7) and a PPV of 12.8% (95% CI 2.9–24.2), while sensitivity was limited to 20.8% (95% CI 5.0–38.9). These findings indicate that although a high Vienna CATS score strongly predicts the absence of VTE in lower-risk patients (NPV 95.6%), the conventional high-risk definition misses the majority of patients who ultimately develop thrombosis.

Across the full range of point thresholds, the score exhibited the expected trade-off between sensitivity and specificity. Lowering the cutoff to ≥ 2 increased sensitivity to 83.3% (95% CI 66.7–96.4) and improved the NPV to 97.6%, at the expense of specificity, which declined to 37.0% (95% CI 32.6–41.6). Conversely, the highest threshold (≥ 7) isolated a very small proportion of the cohort (0.4%) and achieved a PPV of 50.0% with near-perfect specificity of 99.8%, reflecting the score’s ability to discriminate extreme risk levels within the population.

The discriminative capacity of the Vienna CATS score is illustrated by the ROC curve in Fig. 2B, yielding an AUC of 0.672 (95% CI 0.559–0.779). The 95% CI lies entirely above the 0.50 reference line, confirming statistically significant discriminative performance in this sub-cohort. Although the Vienna CATS AUC is numerically higher than the Khorana (0.567), PROTECHT (0.575), and CONKO (0.567) scores, direct statistical comparison is limited because the Vienna CATS analysis was restricted to patients with available biomarker data. Nonetheless, these results suggest that incorporating D-dimer and p-selectin provides additional biological information, enabling more granular stratification of VTE risk, particularly at the extremes of the score distribution.

To compare the discriminative performance of established risk scores, an exploratory AUC analysis using DeLong’s test was performed in the subset of patients with available Vienna CATS scores (*n* = 470), for whom Khorana, PROTECHT, and CONKO scores were also calculated. In this cohort of *n* = 470 patients, the AUC of the Khorana, PROTECHT, and CONKO scores were 0.628 (95% CI: 0.503–0.748), 0.630 (95% CI: 0.504–0.750), and 0.626 (95% CI: 0.505–0.741), respectively.

Pairwise DeLong’s tests comparing the Vienna CATS score with the Khorana, PROTECHT, and CONKO scores showed no statistically significant differences in AUC. Specifically, the comparisons yielded ΔAUCs of 0.044 versus Khorana (Z = 1.59; *p* = 0.111), 0.041 versus PROTECHT (Z = 1.13; *p* = 0.259), and 0.046 versus CONKO (Z = 1.93; *p* = 0.054), indicating that Vienna CATS did not demonstrate a statistically superior discriminative performance relative to the other clinical risk scores in this cohort.

### Cumulative incidence rate

**Fig. 3:**
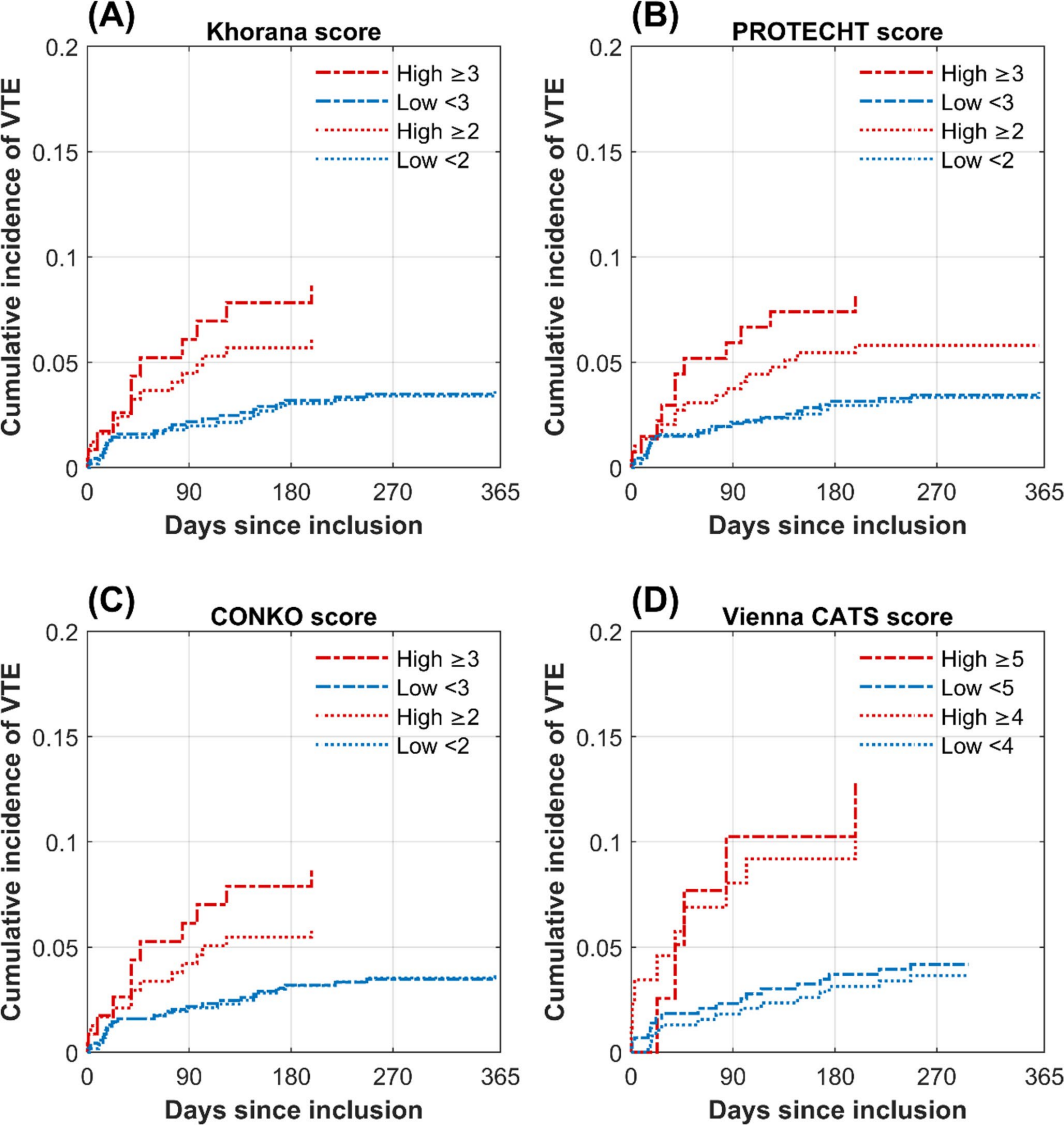
One-year cumulative VTE incidence stratified by traditional and alternative risk score thresholds. Plots based on (**A**) Khorana score, (**B**) PROTECHT score, and (**C**) CONKO score for the full cohort (*n* = 803), and (**D**) Vienna CATS score on subcohort (*n* = 470)

Figure 3 presents the cumulative VTE incidence stratified by traditional and alternative risk score thresholds. At the conventional high-risk cutoff of ≥ 3, the Khorana (Fig. 3A), PROTECHT (Fig. 3B), and CONKO (Fig. 3C) scores effectively identified patient subgroups with markedly elevated VTE incidence compared to low-risk patients. Specifically, high-risk patients according to the Khorana score had a VTE incidence of 9.57% (95% CI 4.62–15.38%), compared with 3.63% (95% CI 2.32–5.14%) in the low-risk group. Comparable patterns were observed for PROTECHT (high-risk: 8.89% [95% CI 4.32–13.97%]; low-risk: 3.59% [95% CI 2.25–5.08%]) and CONKO (high-risk: 9.65% [95% CI 4.55–15.53%]; low-risk: 3.63% [95% CI 2.32–5.09%]). These data indicate that the traditional high-risk definitions concentrate thrombotic events within a relatively small subgroup, approximately tripling the incidence compared to the low-risk population.

Although formal calibration analysis was not performed due to the categorical nature of these risk scores, a comparison between observed and expected event rates provides additional clinical context. In the present cohort, patients classified as high risk by the Khorana score (≥ 3 points) exhibited a VTE incidence of 9.57%, which is modestly higher than the approximately 7% risk originally reported in the derivation study [11]. Similar patterns were observed for the PROTECHT and CONKO scores. These findings suggest that, while the scores retain their ability to stratify relative risk, absolute risk estimates may vary across populations, reinforcing the importance of local validation when applying these models in different clinical settings.

Notably, the observed VTE incidence in the high-risk Khorana group exceeded that reported in the original cohorts, suggesting potential differences in baseline thrombotic risk in our population. This discrepancy further emphasizes the need for regional validation and cautious interpretation of absolute risk estimates when applying these tools in diverse healthcare settings.

Lowering the threshold to ≥ 2 for the clinical-only scores expanded the high-risk population but reduced the contrast between risk strata. At this cutoff, VTE incidence in the high-risk group decreased to 6.50% (95% CI 3.62–9.77%) for Khorana, 6.14% (95% CI 3.57–8.99%) for PROTECHT, and 6.33% (95% CI 3.39–9.54%) for CONKO, while low-risk incidences remained similar (Khorana: 3.59% [95% CI 2.13–5.52%]; PROTECHT: 3.53% [95% CI 2.03–5.18%]; CONKO: 3.71% [95% CI 2.22–5.32%]). This shift illustrates that broadening the cutoff captures more patients in the high-risk category but dilutes the relative enrichment, reducing clinical specificity and precision.

The Vienna CATS score (Fig. 3D), evaluated at its conventional high-risk threshold of ≥ 5, identified the subgroup with the highest observed VTE incidence among all models. High-risk patients at this cutoff experienced a VTE incidence of 12.82% (95% CI 2.94–24.24%), compared to 4.41% (95% CI 2.56–6.44%) in the low-risk group. This enhanced enrichment highlights the incremental prognostic value of incorporating biomarkers such as D-dimer and p-selectin. However, the wide CI reflects the narrow population captured by this stringent threshold.

Using a slightly lower cutoff of ≥ 4, the Vienna CATS score maintained strong discrimination while including a broader patient subset. At this level, high-risk VTE incidence was 11.49% (95% CI 5.19–18.60%), compared with 3.66% (95% CI 1.85–5.63%) for low-risk patients. This intermediate threshold provides a more balanced classification, increasing the number of patients identified as at-risk while slightly reducing the maximum enrichment achieved by the more conservative ≥ 5 cutoff.

### UpSet plots

The UpSet plot (**Figure S1**) provides insight into the internal structure of the Khorana score and its relationship with VTE occurrence. The largest subgroup comprised patients with 0 Khorana points (303 patients, 37.7% of the cohort), among whom 16 VTE events occurred. This finding confirms that a substantial proportion of thrombotic events arise in patients classified as very low risk. The concentration of events within this lowest-risk stratum substantially attenuates the score’s overall discriminative capacity, as a model cannot perform well when many events occur in the reference category.

Approximately 37.5% of patients in our cohort had tumors categorized as high or very-high thrombotic risk, meaning that the majority were classified as having low-risk tumor types. A similar tumor distribution was reported by Overvad et al. [16], in which 59.2% of patients had low-risk tumors and 40.8% had high or very-high risk tumors, according to the Khorana classification.

When examining VTE incidence across Khorana categories, our cohort showed rates of 5.3% in patients with 0 points, 3.1% in the intermediate-risk group (1–2 points), and 9.6% in the high-risk group (≥ 3 points). In contrast, Overvad et al. [16] reported corresponding incidences of 1.47%, 2.83%, and 4.09%, respectively. Notably, in our cohort, the incidence among patients with 0 points exceeded that of the intermediate group, indicating a limited monotonic risk gradient across categories. This pattern weakens the clinical utility of tumor-based stratification and further explains the modest discriminatory performance observed in our cohort and analyses.

Intersectional analysis reveals that hemoglobin and leukocyte count exert a stronger influence on VTE risk than platelets or BMI among patients with very-high-risk tumors plus one component. Anemia (hemoglobin < 10 g/dL) and leukocytosis (> 11 × 10⁹/L) were associated with markedly higher VTE rates (38.5% and 16.7%, respectively) than thrombocytosis (8.7%). In contrast, BMI ≥ 35 kg/m² contributed minimally (0%). In the original derivation study by Khorana et al. [11], multivariable logistic regression demonstrated that hemoglobin had a higher regression coefficient (β = 0.89) than leukocytosis (β = 0.77) and thrombocytosis (β = 0.60), aligned with our findings that anemia may exert a stronger influence on VTE risk than platelet elevation. Notably, BMI showed the highest coefficient (β = 0.90) in that model, which contrasts with our results. Consistent with the prominent role of inflammation, Blix et al. [17] reported that elevated total leukocyte and neutrophil counts measured at least one year prior to cancer diagnosis were associated with a higher future risk of VTE, highlighting the independent contribution of leukocytosis. Similarly, Pabinger et al. [18] suggested that platelet count alone may have limited predictive value and performs better when integrated into RAMs alongside other components. Ha et al. [19] observed a very low prevalence of BMI ≥ 35 kg/m², similar to our cohort, which may explain its limited contribution to risk discrimination. Their study further suggested that lowering the BMI cutoff to ≥ 25 kg/m² improved the prediction of CAT, indicating that the current threshold may underestimate obesity-related risk in certain populations. Overall, despite equal weighting in the Khorana score, these variables likely have complex, potentially nonlinear interactions that traditional linear modeling approaches may not fully capture.

The limited contribution of BMI ≥ 35 kg/m² in our cohort may also reflect the complex and context-dependent relationship between obesity and thrombosis in cancer. While obesity is a well-established risk factor for VTE in the general population, cancer-associated thrombosis appears to be more strongly driven by tumor-related hypercoagulability, systemic inflammation, and endothelial activation. In this setting, laboratory markers reflecting biological perturbation may better capture thrombotic risk than anthropometric measures alone. Furthermore, extreme obesity was infrequent in our cohort, reducing its statistical contribution and suggesting that fixed high BMI thresholds may lack sensitivity in certain populations. These observations support the notion that obesity may interact with, rather than independently dominate, other prothrombotic mechanisms in oncology patients.

The UpSet plot for the PROTECHT score (**Figure S2**) reveals a distribution dominated by low-order intersections, with the majority of the cohort presenting zero or a single risk component. VTE events were infrequently observed and widely dispersed across complex characteristic combinations. Notably, several high-order intersections involving chemotherapy points failed to yield clinical events; for instance, a subset of 7 patients categorized as high risk based on very-high-risk tumor sites and chemotherapy exposure remained VTE-free. Similarly, among seven patients with high-risk tumors and anemia where chemotherapy points were added, only a single VTE event was recorded. Furthermore, in patients lacking tumor-specific risk, the combination of chemotherapy with thrombocytosis and leukocytosis led to the high-risk misclassification of several individuals despite an absence of VTE. Collectively, these patterns indicate that the inclusion of treatment-related variables offers limited incremental discriminatory utility when decoupled from potent baseline biological risk markers.

Within the CONKO model framework, the World Health Organization (WHO) performance status ≥ 2 criterion was met by only 11 patients, none of whom experienced a VTE event (see **Figure S3**). Even among the three patients presenting with the high-risk combination of very-high-risk tumor sites and impaired performance status, no thrombotic events were observed. These results suggest that performance status offered limited incremental prognostic utility within this clinical context.

The UpSet plot for the Vienna CATS score (**Figure S4**) illustrates the impact of integrating D-dimer and soluble P-selectin into the risk stratification framework. The distribution is dominated by low-order intersections, with the largest subgroup (*n* = 118) consisting of patients meeting only the soluble P-selectin threshold (Intersection 1). Complex intersections involving very-high-risk tumor sites, combined with both biomarkers, reached VTE proportions of 20% to 50% (e.g., Intersections 18 and 29).

Overall, the UpSet analyses explain the modest performance of Khorana and its derivatives by demonstrating that high-risk combinations are biologically meaningful but uncommon. The consistent superiority of hemoglobin and leukocytes over platelets and BMI in identifying higher-risk intersections further underscores the limitations of current clinical scores and underscores the need for more biologically informed, nonlinear, or data-driven risk-stratification approaches.

## Discussion

In this prospective cohort of ambulatory cancer patients, the overall VTE incidence was 4.5%, consistent with contemporary real-world data [1, 2]. Comparable rates have been reported across large cohorts, including 4.46% in multiple myeloma [20] and 5.05% in a broad cancer population [21]. Lower incidences have been observed in specific settings, such as patients receiving immune checkpoint inhibitors (2.51%) [22], population-based cohorts (3.3%) [23], and East Asian populations (1.77%) [19], while rates similar to ours have been reported in Hispanic cohorts (4.24%) [24]. These differences likely reflect variations in population characteristics, ethnicity, baseline characteristics, and clinical context.

VTE incidence also varied by tumor type. Higher rates have been reported in lung cancer (9.9% [25] to 18.3% [26]), compared with 6.1% in our cohort. For gynecologic and gastric malignancies, prior studies reported incidences of 6.5% [27] and 6.23% [28], respectively, while our cohort showed a higher rate for gastric cancer (9.8%), comparable to advanced-stage populations [29]. Overall, these findings support a typical 4–5% incidence in unselected ambulatory cohorts, but they also highlight important heterogeneity across tumor types.

The observed incidence may partly reflect the distribution of risk categories, with only 12.6% of patients classified as very high risk and 24.9% as high risk by the Khorana score. Patients who developed VTE exhibited established risk factors, including older age, adenocarcinoma histology, pancreatic cancer, and elevated D-dimer levels, supporting cohort validity. In contrast, several traditional clinical variables did not significantly discriminate risk, underscoring the multifactorial nature of cancer-associated thrombosis in this Brazilian population.

The Khorana, PROTECHT, and CONKO scores showed limited and comparable predictive performance. At conventional thresholds (≥ 3 points), all models demonstrated high specificity but low sensitivity, failing to identify approximately 65–70% of VTE events. These findings align with prior validation studies. Van Es et al. [30] reported modest discrimination (C-statistics ~ 0.5–0.6) and limited separation between risk groups, while Mulder et al. [31] showed high specificity but poor sensitivity, with many events occurring outside high-risk categories. Collectively, these data indicate that clinical-only RAMs provide limited ability to reliably identify patients at the highest risk, functioning primarily to exclude low-risk individuals.

UpSet analyses highlighted key structural limitations of these models. A substantial proportion of VTE events occurred in patients with low or zero scores, contributing to weak discrimination. Importantly, score components contributed unequally to thrombotic risk despite identical weighting. Among patients with very-high-risk tumors plus one additional factor, anemia (hemoglobin < 10 g/dL) and leukocytosis (> 11 × 10⁹/L) were associated with higher VTE rates (38.5% and 16.7%, respectively) compared with thrombocytosis (8.7%), while BMI ≥ 35 kg/m² showed no events (0%). These findings suggest that uniform additive scoring does not adequately capture the biological contribution of individual variables and may partly explain the limited performance of current models. These findings underscore the need to improve the integration of biologically relevant thrombosis biomarkers, many of which are routinely available in clinical practice but are underutilized in current models. Enhanced mathematical representation of these biomarkers may facilitate the development of more accurate, biologically grounded RAMs and strengthen computational modeling of coagulation, where their integration remains limited [32].

Importantly, structural limitations in existing scores may not be fully overcome by simple threshold recalibration, as the issue appears to reside in variable selection and weighting rather than cutoff definition alone. Advanced modeling approaches may offer a path forward. These include machine learning models [33, 34], VTE prediction by mechanistic computational models [35] and hybrid models [35–37]. Integrating insights from these strategies with expert knowledge and existing risk scores may enable the development of risk scores that incorporate a broader set of variables while remaining simple, nonlinear, yet manageable, clinically applicable, and more accurate for VTE risk assessment.

Adjusting score thresholds did not yield meaningful clinical improvement, as substantial losses in specificity offset gains in sensitivity. This indicates that limitations are primarily structural, related to variable selection and weighting, rather than threshold definition alone.

In contrast, Vienna CATS score demonstrated higher discrimination, though not statistically superior, supporting the value of biomarker-based approaches. The incorporation of D-dimer and soluble Pe-selectin likely improves risk estimation by capturing tumor-related hypercoagulability and endothelial activation, processes not reflected in clinical variables alone. Although sensitivity remained modest, higher score thresholds were associated with substantial risk enrichment, suggesting potential utility in identifying patients at particularly elevated risk.

Cumulative incidence analyses further illustrated the trade-off between risk concentration and population coverage: stricter thresholds increased absolute risk but applied to smaller subgroups, whereas broader thresholds diluted discrimination.

Overall, our findings underscore the persistent limitations of current clinical RAMs for cancer-associated VTE. Notably, approximately one-third of VTE events occurred in patients classified as low risk, highlighting a critical limitation of these models and their inability to reliably identify patients in whom thromboprophylaxis can be safely withheld. Consistently, the low sensitivity observed indicates that current high-risk definitions do not capture a substantial proportion of at-risk patients. These findings suggest that using RAMs alone to guide thromboprophylaxis decisions may be inadequate in this setting. While these models may still help identify lower-risk populations, their clinical utility for directing preventive strategies remains limited. Improved prediction will likely require incorporating additional variables, including novel biomarkers, evaluating alternative predictor thresholds and applying more flexible approaches, such as nonlinear weighting of risk factors. Future research should focus on developing and validating multimodal, locally applicable models to support individualized thromboprophylaxis in routine clinical practice.

This study has several limitations that should be acknowledged. First, although the overall cohort size was substantial and the observed VTE incidence was consistent with prior literature, the absolute number of VTE events was relatively low (*n* = 36), representing a key limitation for external validation studies. Rule-of-thumb recommendations in the methodological literature typically suggest a minimum of 100 outcome events (and 100 non-events) to ensure stable estimation of predictive performance in binary risk models [38–40]. The number of events in the present study falls below this threshold. This limited event rate not only reduces statistical power for subgroup and intersectional analyses but also has important implications for the stability and precision of global performance metrics, including AUC estimates, across all evaluated models. As a result, CIs around discrimination measures are relatively wide, and comparisons between models should be interpreted with caution. In particular, the absence of statistically significant differences in pairwise comparisons may reflect limited statistical power rather than true equivalence in predictive performance. For example, the borderline result observed in the comparison between Vienna CATS and CONKO (*p* = 0.054) may be attributable to insufficient event counts and should therefore not be overinterpreted as evidence of similar model performance.

Second, this study represents a single-country cohort derived from a limited number of institutions within a single academic public healthcare system in southeastern Brazil. As such, it reflects a single-region, institutionally based population and may not fully capture the substantial regional, socioeconomic, and ethnic heterogeneity of the broader Brazilian population. Variations in cancer epidemiology, healthcare access, and treatment practices across different regions of Brazil may influence both baseline thrombotic risk and the performance of risk assessment models. Consequently, caution is warranted when extrapolating these findings to settings similar to them. Future studies, including cohorts from diverse geographic regions and healthcare systems across Brazil, will be essential to establish the robustness and national generalizability of these results.

Third, asymptomatic thrombotic events were not systematically screened, raising the possibility of underestimation of true incidence. Additionally, biomarker-based analyses were performed within a defined subcohort, potentially introducing selection bias despite the prospective design.

Fourth, the evaluated risk scores were applied across all tumor types in this cohort. However, some malignancies, such as multiple myeloma and central nervous system tumors, were not included in the original derivation of certain models (e.g., the Khorana score) and may not be adequately represented by them. Consequently, model performance could differ in cohorts restricted to tumor types for which these scores were originally developed. In the present study, no exclusions based on tumor site were applied, reflecting a real-world clinical population but potentially contributing to the observed limitations in predictive performance.

Finally, risk assessment was based on baseline variables, and dynamic changes in laboratory markers, treatment exposure, or disease progression were not incorporated into the models.

Future research should focus on the development and validation of multimodal risk prediction strategies that integrate clinical characteristics, longitudinal biomarkers, and potentially mechanistic or data-driven modeling approaches. Dynamic risk assessment frameworks that account for temporal variation in thrombotic risk during cancer treatment may improve prediction accuracy beyond static baseline scores. Large, externally validated, and ethnically diverse cohorts will be essential to determine generalizability and clinical applicability. Ultimately, prospective interventional studies are required to determine whether improved risk stratification translates into safer, more effective personalized thromboprophylaxis strategies in routine oncologic care.

## Conclusion

In this prospective Brazilian cohort, the Khorana, PROTECHT, and CONKO scores demonstrated limited discrimination and low sensitivity for identifying patients who developed VTE, missing the majority of events at conventional thresholds. Notably, a substantial proportion of VTE events occurred among patients classified as low risk, highlighting the inability of these models to reliably exclude patients from thromboprophylaxis. The Vienna CATS score showed statistically significant discrimination compared to chance, but was not superior to clinical-only scores in direct comparisons. Overall, current risk assessment models primarily stratify relative risk but have limited ability to accurately identify patients at high risk or to safely guide thromboprophylaxis decisions when used alone. These findings support the need for improved approaches, including the incorporation of additional variables and biomarkers, the evaluation of alternative thresholds, the application of more flexible strategies such as nonlinear modeling, and the development of locally validated prediction tools.

## Electronic Supplementary Material

Below is the link to the electronic supplementary material.

Supplementary Material 1

Supplementary Material 2

Supplementary Material 3

Supplementary Material 4

Supplementary Material 5

Supplementary Material 6

## Data Availability

The datasets generated and/or analyzed during the current study are not publicly available due to ethical and privacy restrictions involving personal data. Access to the data may be granted upon reasonable request to the corresponding author, subject to approval by the institutional ethics committee and compliance with applicable regulations.

## Abbreviations

AUC: Area Under the Curve
BMI: Body Mass Index
CAAE: Certificate of Presentation for Ethical Consideration (Certificado de Apresentação para Apreciação Ética)
CAT: Cancer-Associated Thrombosis
CAISM: Women’s Comprehensive Health Care Center
CDT: Center for Thromboembolic Diseases
CI: Confidence Interval
DVT: Deep Vein Thrombosis
FEU: Fibrinogen Equivalent Units
IQR: Interquartile range
NPV: Negative Predictive Value
PE: Pulmonary Embolism
PPV: Positive Predictive Value
RAM: Risk Assessment Model
REDCAP: Research Electronic Data Capture
ROC: Receiver Operating Characteristic
TRIPOD: Transparent Reporting of a Multivariable Prediction Model for Individual Prognosis or Diagnosis
UNICAMP: University of Campinas
VTE: Venous Thromboembolism
WHO: World Health Organization
