# Supplementary material for: External validation of established clinical risk scores for cancer-associated venous thromboembolism in a Brazilian registry
Source: J Thromb Thrombolysis. 2026 May 18;59(6):1411–30. doi: 10.1007/s11239-026-03303-6 (PMC13447457; doi:10.1007/s11239-026-03303-6)
Supplement: Supplementary file 1 [file 11239_2026_3303_MOESM5_ESM.docx]

**Table S1** Comparison of baseline demographic, clinical, tumor-related, and laboratory characteristics according to availability of D-dimer and soluble p-selectin measurements in the overall cohort.

| **Characteristic** | **All patients (N = 803)** | **D-dimer and**  **P-selection available**  **(n = 470)** | **D-dimer and**  **p-selectin non available**  **(n = 333)** | **P** **†** |
| --- | --- | --- | --- | --- |
| **Age, years, median (25th-75th percentile)** | 63.0 (53.0–71.0) | 62.5 (53.0–71.0) | 64.0 (54.0–71.0) | 0.417 |
| **Female sex, % (n)** | 50.9% (409) | 50.4% (237) | 51.4% (171) | 0.830 |
| **Body mass index, kg/m^2^, median (25th-75th percentile)** | 24.9 (21.6–28.8) | 25.1 (22.0–28.9) | 24.6 (21.3–28.7) | 0.228 |
| **Statin use, % (n)** | 17.4% (140) | 16.8% (79) | 18.3% (61) | 0.637 |
| **Antidiabetic medication, % (n)** | 15.8% (127) | 16.0% (75) | 15.9% (53) | 1.000 |
| **Hormone therapy use, % (n)** |  |  |  |  |
| Current use (oral contraceptives, injectable contraceptives, or hormone replacement therapy) | 48.7% (391) | 49.6% (233) | 47.7% (159) | 0.617 |
| No use | 23.4% (188) | 26.2% (123) | 19.5% (65) | **0.034** |
| **Previous thrombosis, % (n)** | 6.5% (52) | 6.4% (30) | 6.6% (22) | 0.886 |
| **Surgery >30 min with anesthesia, % (n)** | 71.7% (576) | 69.1% (325) | 75.1% (250) | 0.068 |
| **Any immobilization, % (n)** | 18.9% (152) | 17.2% (81) | 21.0% (70) | 0.199 |
| **World Health Organization functional status ≥ 2, % (n)** | 2.2% (18) | 2.3% (11/469) | 2.1% (7) | 1.000 |
| **Education, % (n)** |  |  |  |  |
| No formal education | 10.6% (85) | 7.4% (35) | 14.7% (49) | **<0.001** |
| Primary education | 53.7% (431) | 54.5% (256) | 52.9% (176) | 0.667 |
| Secondary education | 26.3% (211) | 28.9% (136) | 22.5% (75) | **0.042** |
| Higher education | 9.5% (76) | 9.1% (43) | 9.9% (33) | 0.715 |
| **Lifestyle factors, % (n)** |  |  |  |  |
| Current smoker | 11.1% (89) | 11.7% (55) | 10.2% (34) | 0.569 |
| Former smoker | 36.0% (289) | 33.0% (155) | 40.2% (134) | **0.037** |
| Never smoker | 52.9% (425) | 55.3% (260) | 49.5% (165) | 0.115 |
| Current alcohol use | 3.7% (30) | 4.3% (20) | 3.0% (10) | 0.451 |
| Former alcohol use | 13.0% (104) | 12.3% (58) | 14.1% (47) | 0.460 |
| Never alcohol use | 83.3% (669) | 83.4% (392) | 82.9% (276) | 0.849 |
| **Chemotherapy regimen % (n)** |  |  |  |  |
| Platinum-based chemotherapy | 15.8% (127) | 15.5% (73) | 15.9% (53) | 0.922 |
| Gemcitabine-based chemotherapy | 1.2% (10) | 1.5% (7) | 0.9% (3) | 0.535 |
| Platinum plus gemcitabine chemotherapy | 7.6% (61) | 7.0% (33) | 8.1% (27) | 0.588 |
| **Tumor histology, % (n)** |  |  |  |  |
| Adenocarcinoma | 39.1% (314) | 40.4% (190) | 37.2% (124) | 0.379 |
| Carcinoma | 34.0% (273) | 28.9% (136) | 41.1% (137) | **<0.001** |
| Germ cell tumor | 0.2% (2) | 0.0% (0) | 0.6% (2) | 0.172 |
| Glioma | 1.9% (15) | 2.6% (12) | 0.9% (3) | 0.114 |
| Hodgkin lymphoma | 2.5% (20) | 2.8% (13) | 2.1% (7) | 0.649 |
| Non-Hodgkin lymphoma | 8.2% (66) | 9.8% (46) | 6.0% (20) | 0.067 |
| Melanoma | 1.1% (9) | 0.6% (3) | 1.8% (6) | 0.174 |
| Multiple myeloma | 5.0% (40) | 6.6% (31) | 2.7% (9) | **0.013** |
| Trophoblastic neoplasia | 0.1% (1) | 0.0% (0) | 0.3% (1) | 0.415 |
| Sarcoma | 2.4% (19) | 3.2% (15) | 1.2% (4) | 0.097 |
| Neuroendocrine tumor | 0.7% (6) | 0.6% (3) | 0.9% (3) | 0.696 |
| Other histologies | 4.7% (38) | 4.5% (21) | 5.1% (17) | 0.737 |
| **Metastatic, % (n)** |  |  |  |  |
| Yes | 4.1% (33) | 3.4% (16) | 5.1% (17) | 0.279 |
| No | 80.0% (642) | 77.4% (364) | 83.5% (278) | **0.040** |
| Not reported | 15.9% (128) | 19.1% (90) | 11.4% (38) | **0.003** |
| **Tumor location, % (n)** |  |  |  |  |
| Pancreas | 3.9% (31) | 4.5% (21) | 3.0% (10) | 0.354 |
| Stomach | 8.7% (70) | 8.7% (41) | 8.7% (29) | 1.000 |
| Lung / Pleura | 6.1% (49) | 4.7% (22) | 8.1% (27) | 0.052 |
| Ovarian | 0.7% (6) | 1.1% (5) | 0.3% (1) | 0.409 |
| Uterine (corpus) | 0.6% (5) | 0.4% (2) | 0.9% (3) | 0.654 |
| Cervical | 1.6% (13) | 1.9% (9) | 1.2% (4) | 0.574 |
| Testicular | 1.0% (8) | 0.6% (3) | 1.5% (5) | 0.287 |
| Lymphoma (non-solid) | 10.6% (85) | 12.3% (58) | 8.1% (27) | 0.062 |
| Renal | 2.5% (20) | 1.9% (9) | 3.3% (11) | 0.253 |
| Bladder | 1.7% (14) | 1.7% (8) | 1.8% (6) | 1.000 |
| Breast | 13.7% (110) | 10.9% (51) | 17.4% (58) | **0.009** |
| Colon | 6.5% (52) | 7.7% (36) | 4.8% (16) | 0.112 |
| Rectum | 9.5% (76) | 11.1% (52) | 7.2% (24) | 0.068 |
| Esophagus | 5.7% (46) | 5.1% (24) | 6.6% (22) | 0.441 |
| Liver | 2.9% (23) | 2.6% (12) | 3.6% (12) | 0.407 |
| Small intestine | 2.7% (22) | 2.3% (11) | 3.3% (11) | 0.511 |
| Bone and cartilage | 2.7% (22) | 3.6% (17) | 1.5% (5) | 0.081 |
| Soft tissue | 0.2% (2) | 0.4% (2) | 0.0% (0) | 0.514 |
| Skin | 1.2% (10) | 0.9% (4) | 1.8% (6) | 0.334 |
| Central nervous system | 2.0% (16) | 2.8% (13) | 0.9% (3) | 0.074 |
| Head and neck | 4.2% (34) | 2.6% (12) | 6.6% (22) | **0.007** |
| Bone marrow | 5.0% (40) | 6.6% (31) | 2.7% (9) | **0.013** |
| Mediastinum | 0.1% (1) | 0.0% (0) | 0.3% (1) | 0.415 |
| Peritoneum | 0.5% (4) | 0.4% (2) | 0.6% (2) | 1.000 |
| Prostate | 2.9% (23) | 3.2% (15) | 2.4% (8) | 0.668 |
| Adrenal | 0.5% (4) | 0.2% (1) | 0.9% (3) | 0.313 |
| Biliary tract | 1.0% (8) | 1.1% (5) | 0.9% (3) | 1.000 |
| Anal canal | 0.4% (3) | 0.4% (2) | 0.3% (1) | 1.000 |
| Penis | 0.2% (2) | 0.2% (1) | 0.3% (1) | 1.000 |
| Thymus / lymph node / spleen | 0.4% (3) | 0.2% (1) | 0.6% (2) | 0.573 |
| Ureters / renal pelvis | 0.1% (1) | 0.0% (0) | 0.3% (1) | 0.415 |
| **Laboratory parameters, median (25th–75th percentile)** |  |  |  |  |
| Platelet count (×10⁹/L) | 271.0 (210.0–346.8) | 267.0 (205.0–349.0) | 274.0 (221.0–344.0) | 0.419 |
| Hemoglobin (g/dL) | 12.5 (10.8–13.7) | 12.4 (10.8–13.7) | 12.7 (10.8–13.7) | 0.494 |
| Total white blood cell count (×10⁹/L) | 7.7 (5.9–9.6) | 7.7 (6.1–9.6) | 7.5 (5.8–9.6) | 0.436 |
| Red blood cell count (×10¹²/L) | 4.3 (3.8–4.7) | 4.3 (3.7–4.8) | 4.3 (3.9–4.7) | 0.651 |
| Hematocrit (%) | 38.0 (33.4–41.5) | 37.8 (33.2–41.7) | 38.0 (33.5–41.3) | 0.715 |
| Absolute neutrophil count (×10⁹/L) | 4.7 (3.5–6.4) | 4.7 (3.4–6.4) | 4.7 (3.5–6.4) | 0.926 |
| Red cell distribution width (%) | 14.1 (13.3–15.4) | 14.0 (13.2–15.3) | 14.2 (13.3–15.4) | 0.125 |
| **Antiplatelet and Anticoagulant prophylaxis, n (%)** |  |  |  |  |
| Aspirin | 3.0% (24) | 4.0% (19) | 1.5% (5) | 0.056 |
| Clopidogrel | 0.2% (2) | 0.2% (1) | 0.3% (1) | 1.000 |
| Enoxaparin | 11.5% (92) | 12.6% (59) | 9.9% (33) | 0.262 |
| Rivaroxaban | 1.1% (9) | 1.1% (5) | 1.2% (4) | 1.000 |
| Unfractionated heparin | 1.6% (13) | 1.3% (6) | 2.1% (7) | 0.403 |
| No pharmacologic antiplatelet and anticoagulation | 82.6% (663) | 80.9% (380) | 85.0% (283) | 0.132 |
| **Outcome*: Venous thromboembolism % (n)** | 4.5% (36) | 5.1% (24) | 3.6% (12) | 0.387 |

*VTE includes objectively confirmed venous thromboembolism events during follow-up.

†P values were calculated using the Mann–Whitney U test for continuous variables and Fisher’s exact test for categorical variables. A two-sided P < 0.05 was considered statistically significant.
