# Supplementary material for: External validation of established clinical risk scores for cancer-associated venous thromboembolism in a Brazilian registry
Source: J Thromb Thrombolysis. 2026 May 18;59(6):1411–30. doi: 10.1007/s11239-026-03303-6 (PMC13447457; doi:10.1007/s11239-026-03303-6)
Supplement: Supplementary file 2 [file 11239_2026_3303_MOESM6_ESM.docx]

Table S2: TRIPOD checklist for reporting of the external validation of VTE risk prediction models in a prospective Brazilian cancer cohort

| **Section/Topic** | **Item** | **Checklist item** | **Where in paper / description** |
| --- | --- | --- | --- |
| Title and abstract |  |  |  |
| Title | 1 | Identify the study as developing and/or validating a multivariable prediction model,  the target population, and the outcome to be predicted | Title: “External Validation … Clinical Risk Scores … Cancer-Associated Venous Thromboembolism … Brazilian registry” |
| Abstract | 2 | Provide a summary of objectives, study design, setting, participants, sample size,  predictors, outcome, statistical analysis, results, and conclusions | Abstract provides a comprehensive summary of the study, including:   - Objective (external validation of Khorana, PROTECHT, CONKO, Vienna CATS) - Design (prospective cohort) - Setting/participants (Brazilian tertiary-care registry; n = 803; subcohort n = 470) - Predictors (baseline variables from original models) - Outcome (12-month symptomatic VTE) - Analysis (AUC, bootstrap CI, DeLong test) - Results (36/803 events; AUC ~0.57; Vienna CATS 0.672) - Conclusion (limited discrimination) |
| Introduction |  |  |  |
| Background and objectives | 3a | Explain the medical context (including whether diagnostic or prognostic) and rationale  for developing or validating the multivariable prediction model, including references  to existing models | Section 1. Introduction:   - Defines CAT as a prognostic problem - Describes limitations of existing RAMs (low sensitivity, heterogeneity) - Highlights lack of Brazilian validation data |
|  | 3b | Specify the objectives, including whether the study describes the development or  validation of the model, or both | Section 1. Introduction:   - Explicit objective to externally validate and compare four RAMs |
| Methods |  |  |  |
| Source of data | 4a | Describe the study design or source of data (for example, randomised trial, cohort,  or registry data), separately for the development and validation data sets, if applicable | Section 2.1. Study population:   - Prospective multicenter cohort (external validation study) - Registry-based data from tertiary-care institutions |
|  | 4b | Specify the key study dates, including start of accrual; end of accrual; and, if applicable,  end of follow-up | Section 2.1. Study population:   - Recruitment: July 2016 – July 2020 - Follow-up: up to 12 months |
| Participants | 5a | Specify key elements of the study setting (for example, primary care, secondary care,  general population) including number and location of centres | Section 2.1. Study population:   - Tertiary-care oncology/hematology centers - Three institutions in Campinas, Brazil |
|  | 5b | Describe eligibility criteria for participants | Section 2.1. Study population:   - Inclusion: adult ambulatory cancer patients initiating chemotherapy - Exclusion: recent VTE, therapeutic anticoagulation |
|  | 5c | Give details of treatments received, if relevant | Section 2.1. Study population:   - Patients enrolled prior to initiation of a new chemotherapy regimen - Treatment exposure not modeled as a predictor - Antiplatelet therapy permitted |
| Outcome | 6a | Clearly define the outcome that is predicted by the prediction model, including how  and when assessed | Section 2.2. Outcome Definition and Adjudication:   - Primary outcome: objectively confirmed symptomatic or fatal VTE within 12 months - Diagnostic methods specified (ultrasound, CT angiography, ventilation–perfusion lung scintigraphy) |
|  | 6b | Report any actions to blind assessment of the outcome to be predicted | Section 2.2. Outcome Definition and Adjudication:   - No formal blinding - Outcome based on objective imaging reports |
| Predictors | 7a | Clearly define all predictors used in developing the multivariable prediction model,  including how and when they were measured | Section 2.1. Study population and Section 2.3. Risk Score Assessment:   - All predictors defined according to original RAMs - Measured at baseline prior to chemotherapy - No transformation or recalibration |
|  | 7b | Report any actions to blind assessment of predictors for the outcome and other  predictors | Section 2.3. Risk Score Assessment:   - No formal blinding - Predictors collected prospectively before outcome occurrence |
| Sample size | 8 | Explain how the study size was arrived at | Section 2.4. Statistical analyses:   - Determined by available cohort - No formal calculation performed |
| Missing data | 9 | Describe how missing data were handled (for example, complete-case analysis,  single imputation, multiple imputation) with details of any imputation method | Section 2.4. Statistical analyses:   - No missing baseline predictor or outcome data - No imputation required |
| Statistical analysis methods | 10a | Describe how predictors were handled in the analyses | Section 2.3. Risk Score Assessment and Section 2.4. Statistical analyses:   - Predictors used exactly as defined in original models - No transformation or reweighting |
|  | 10b | Specify type of model, all model-building procedures (including any predictor selection),  and method for internal validation | Not applicable (external validation only) |
|  | 10c | For validation, describe how the predictions were calculated | Section 2.3. Risk Score Assessment:   - Scores computed using original definitions - Predictions derived as categorical risk classifications based on score thresholds - Risk thresholds: Khorana/PROTECHT/CONKO: ≥3 (primary), ≥2 (secondary) - Risk thresholds: Vienna CATS: ≥5 (primary), ≥4 (secondary) |
|  | 10d | Specify all measures used to assess model performance and, if relevant, to compare  multiple models | Section 2.4. Statistical analyses:   - Discrimination: ROC curves, AUC - Classification: sensitivity, specificity, PPV, NPV - Uncertainty: bootstrap (10,000 iterations) - Model comparison: DeLong test - Time-to-event: cumulative incidence curves |
|  | 10e | Describe any model updating (for example, recalibration) arising from the validation,  if done | Section 2.4. Statistical analyses:   - No model updating or recalibration performed |
| Risk groups | 11 | Provide details on how risk groups were created, if done | Section 2.3. Risk Score Assessment:   - Defined using pre-specified thresholds from original models - Alternative thresholds evaluated |
| Development v validation | 12 | For validation, identify any differences from the development data in setting, eligibility  criteria, outcome, and predictors | - Not applicable |
| Results |  |  |  |
| Participants | 13a | Describe the flow of participants through the study, including the number of participants  with and without the outcome and, if applicable, a summary of the follow-up time. A  diagram may be helpful | Section 3.1. Population characteristics; Section 3.2. Follow-up and outcomes; Figure 1:   - Flow diagram provided (Figure 1) - Final cohorts: n = 803 (clinical RAMs) - Final cohorts: n = 470 (Vienna CATS) - Events: 36/803 (4.5%) - Median time-to-event: 75 days |
|  | 13b | Describe the characteristics of the participants (basic demographics, clinical features,  available predictors), including the number of participants with missing data for  predictors and outcome | Section 3.1. Population characteristics; Tables 1, 2, S1:   - Detailed baseline characteristics (Tables 1, 2, S1) - Includes demographics, clinical variables, tumor type, laboratory markers - Stratified by VTE status |
|  | 13c | For validation, show a comparison with the development data of the distribution of  important variables (demographics, predictors and outcome) | Section 4. Discussion   - Indirect comparison with development and validation cohorts through: - Reported VTE incidence ranges across studies - Reported AUC values (~0.5–0.6 for clinical RAMs) - No formal tabulated comparison of predictor distributions |
| Model development | 14a | Specify the number of participants and outcome events in each analysis | Not applicable |
|  | 14b | If done, report the unadjusted association between each candidate predictor and  outcome | Not applicable |
| Model specification | 15a | Present the full prediction model to allow predictions for individuals (that is, all  regression coefficients, and model intercept or baseline survival at a given time point) | Not applicable |
|  | 15b | Explain how to use the prediction model | Not applicable |
| Model performance | 16 | Report performance measures (with CIs) for the prediction model | Section 3.3. Performance of Khorana, PROTECHT, and CONKO; Section 3.4. Performance of Vienna CATS; Tables 3 and Figures 2A–2B:   - AUC with 95% CI for all models - Sensitivity, specificity, PPV, NPV at thresholds - ROC curves and DeLong comparisons |
| Model updating | 17 | If done, report the results from any model updating (that is, model specification, model  performance) | Not applicable |
| Discussion |  |  |  |
| Limitations | 18 | Discuss any limitations of the study (such as nonrepresentative sample, few events  per predictor, missing data) | Section 4. Discussion (limitations paragraphs):   - Low number of events (n = 36) → limited power and precision - Single-region cohort → limited generalizability - No screening for asymptomatic VTE - Biomarker subcohort → potential selection bias - Static baseline predictors - Some tumor types not represented in derivation models |
| Interpretation | 19a | For validation, discuss the results with reference to performance in the development  data, and any other validation data | Section 4. Discussion:   - Compared with prior validation studies (Van Es, Mulder) - Similar findings: modest discrimination (AUC ~0.5–0.6), low sensitivity |
|  | 19b | Give an overall interpretation of the results, considering objectives, limitations, results  from similar studies, and other relevant evidence | Section 4. Discussion:   - Clinical RAMs have limited ability to identify high-risk patients - Structural limitations (variable weighting, biology not captured) - Biomarkers improve but do not solve prediction |
| Implications | 20 | Discuss the potential clinical use of the model and implications for future research | Section 4. Discussion:  Current models mainly useful to identify low-risk patients  Limited utility for guiding thromboprophylaxis  Future directions: Multimodal models, biomarkers + nonlinear approaches, and dynamic risk prediction |
| Other information |  |  |  |
| Supplementary information | 21 | Provide information about the availability of supplementary resources, such as study  protocol, Web calculator, and data sets | Supplementary materials:   - Supplementary tables (Table S1, Table S2) and UpSet plots (Figures S1–S4) - No protocol or web calculator reported |
| Funding | 22 | Give the source of funding and the role of the funders for the present study | Funding statement section:   - Funding sources and roles reported |
